# The Gut-Immune-Brain Axis in Autism Spectrum Disorders; A Focus on Amino Acids

**DOI:** 10.3389/fendo.2019.00247

**Published:** 2019-04-16

**Authors:** Joris H. J. van Sadelhoff, Paula Perez Pardo, Jiangbo Wu, Johan Garssen, Jeroen van Bergenhenegouwen, Astrid Hogenkamp, Anita Hartog, Aletta D. Kraneveld

**Affiliations:** ^1^Division of Pharmacology, Utrecht Institute for Pharmaceutical Sciences, Faculty of Science, Utrecht University, Utrecht, Netherlands; ^2^Danone Nutricia Research, Utrecht, Netherlands; ^3^Laboratory of Neuroimmunology, Department of Symptom Research, The University of Texas MD Anderson Cancer Center, Houston, TX, United States; ^4^Veterinary Pharmacology, Institute for Risk Assessment Studies, Faculty of Veterinary Sciences, Utrecht University, Utrecht, Netherlands

**Keywords:** autism spectrum disorder, amino acids, mammalian target of rapamycin, nutritional intervention, gut-immune-brain axis

## Abstract

Autism spectrum disorder (ASD) is a range of neurodevelopmental conditions that affect communication and social behavior. Besides social deficits, systemic inflammation, gastrointestinal immune-related problems, and changes in the gut microbiota composition are characteristic for people with ASD. Animal models showed that these characteristics can induce ASD-associated behavior, suggesting an intimate relationship between the microbiota, gut, immune system and the brain in ASD. Multiple factors can contribute to the development of ASD, but mutations leading to enhanced activation of the mammalian target of rapamycin (mTOR) are reported frequently. Hyperactivation of mTOR leads to deficits in the communication between neurons in the brain and to immune impairments. Hence, mTOR might be a critical factor linking the gut-brain-immune axis in ASD. Pharmacological inhibition of mTOR is shown to improve ASD-associated behavior and immune functions, however, the clinical use is limited due to severe side reactions. Interestingly, studies have shown that mTOR activation can also be modified by nutritional stimuli, in particular by amino acids. Moreover, specific amino acids are demonstrated to inhibit inflammation, improve gut barrier function and to modify the microbiota composition. In this review we will discuss the gut-brain-immune axis in ASD and explore the potential of amino acids as a treatment option for ASD, either via modification of mTOR activity, the immune system or the gut microbiota composition.

## The Gut-Brain Axis in Autism Spectrum Disorder

Autism spectrum disorder (ASD) is a spectrum of neurological disorders that become apparent in early childhood and last throughout a person's life. This spectrum of disorders is characterized by varying levels of deficits in social behavior and communication, as well as stereotypical behaviors (1). Estimates of the prevalence of ASD vary widely among different studies, largely attributable to differences in diagnostic criteria, the ages and gender of the children screened, as well as the geographical location (2). Meta-analyses comprising studies in different geographical locations indicate a global incidence of ASD between 0.6 and 0.7% (3, 4). Over the past decades, the reported global prevalence has increased markedly (4, 5). Studies suggest that part of this increase is attributable to changes in diagnostic criteria, an increased awareness and the ability to diagnose at a younger age (3, 6). However, these changes alone are not sufficient to explain the rise in ASD prevalence. Other factors, including prenatal, natal and postnatal environmental factors like diet, intestinal microbiota, and immunological triggers are likely to also contribute to this increase (7–9).

In addition to behavioral deficits, autistic individuals often present with mild systemic inflammation and gastrointestinal disorders (10, 11). Although current epidemiological data is limited, a recent meta-analysis confirms that children with ASD experience four times more gastrointestinal symptoms than control groups and that gastrointestinal symptoms may identify a unique subgroup of children with ASD (12). Moreover, numerous studies showed a link between immune disturbances and ASD (11, 13, 14). Although research into the relation between immune disturbances and ASD is still in its early phases, studies suggest that many different aspects of the immune system are involved (15–17). For instance, ASD has been associated with a deregulated activation of microglia and astroglia (the “immune-like” cells in the brain) (18, 19), autoimmunity (20), increased T cell activation (21), deregulated innate immune functioning (11) and mutations in genes controlling the functioning of immune cells (11, 22, 23). The deregulated immune functions in people with ASD are reflected by abnormal cytokine levels in their body fluids. More specifically, levels of pro-inflammatory and allergy-associated type 2 helper T (Th2) cell-derived cytokines (e.g., TNFα, IL-4, IL-5, IL-6, IL-8) are shown to be upregulated in either cerebrospinal fluid, plasma or serum of people with ASD (21), whereas levels of regulatory cytokines IL-10 and TGF-β are downregulated (24–26). Multiple studies investigated the relation between the aberrant cytokine levels and autism. One example is a study performed by Hashim et al. (27). Here, it was shown that serum levels of TGF-β negatively correlated with the severity of autistic symptoms. A similar negative correlation was found between the severity of autism and the frequency of regulatory T (Treg) cells in blood, which appears to be lower in autistic individuals (28). As TGF-β plays a critical role in Treg cell development (29), there might be a causal relation between lower TGF-β levels, lower Treg levels and autism severity. Besides its role in the development of Treg cells, TGF-β is also indicated to play an important role in brain development (30, 31). More specifically, TGF-β regulates survival and differentiation of neurons and directs the growth of synapses, as observed in various invertebrate models (30, 31). Hence, a lack of TGF-β could contribute to dysregulated brain functioning, representing another mechanism by which aberrant TGF-β levels could be involved in the development of autistic phenotypes observed in autistic patients. The presence of a relation between abnormal cytokine levels in autistic individuals and autism severity is further corroborated by two large studies in children with ASD (32, 33). In these studies it was demonstrated that the increase of a broad range of cytokines including pro-inflammatory, Th2 as well as type 1 helper T (Th1) cell-derived cytokines and chemokines correlates with aberrant (autistic) behavior and impaired development (32, 33).

**Figure 1:**
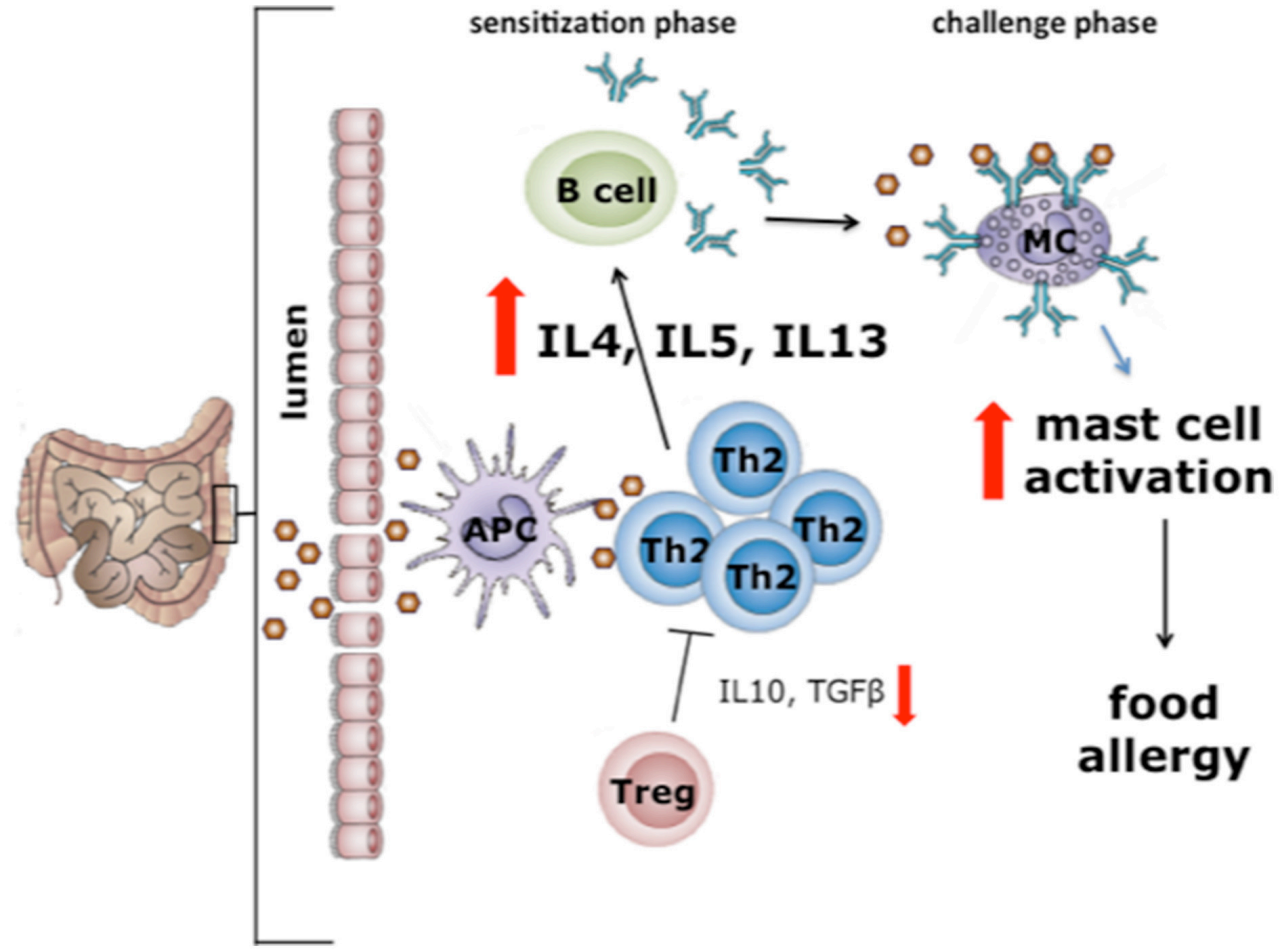
In the first phase of allergy or type 1 hypersensitivity reactions sensitization to the food allergen takes place (sensitization phase). During this phase, allergens cross the intestinal mucosal barrier and are presented via MHC-II to naïve T cells that develop into type 2 helper T (Th2) cells. Th2 cells induce the plasma cells to produce IgE antibodies (37), after which IgE binds to the FcεRI receptor on mast cells. In the challenge phase, upon a second exposure to the same allergen, cross-linking of mast cell-bound IgE occurs. In turn, this leads to mast cell degranulation, a process resulting in the release of several mediators including histamine, cytokines and tryptases. The release of these mediators is the direct cause of most of the allergic intestinal symptoms, including nausea, vomiting, cramps, and diarrhea (38). With permission of Dr J. Wu.

The exact mechanisms underlying the increased production of pro-inflammatory cytokines and allergy-associated Th2 cytokines in autistic individuals are yet to be elucidated. However, a potential explanation of these findings might be sought in the observation that allergy, especially food allergy, and eosinophilic esophagitis are often reported in ASD patients (34, 35). A food-induced allergic disease is an unfavorable immune reaction that can occur on exposure to specific food components, particularly proteins (36). Classically, food allergy is regarded as a type 1 hypersensitivity, i.e., an IgE-mediated immune response to harmless food proteins (Figure 1).

## The Immune-Brain Axis in ASD With a Focus on Food Allergy

It has been shown from parental reports that food allergies are observed more often in autistic individuals than in the general population (39–41). This differential occurrence of food allergies might be even more evident than reported, since impaired communication by autistic individuals could lead to underdiagnoses (39). Multiple studies investigated the relation of food allergies with ASD. For instance, it has been reported that autistic children have higher serum levels of IgA, IgG, and IgM specific for cow's milk proteins compared to healthy controls (42, 43). Interestingly, Lucarelli et al. showed that autistic children on a cow's milk free diet showed improved behavioral symptoms (43). Vice versa, challenging autistic individuals with cow's milk led to an increase in hallmark behaviors associated with ASD, providing evidence for a relation between food allergic reactions and behavior (43). Preclinical studies provide further evidence for a relation between food allergies and ASD, as cow's milk allergic (CMA) mice show autistic-like behavior such as reduced social interaction, increased repetitive behavior, disturbed spatial memory and reduced exploration behavior (44). The changes in behavior of CMA mice were associated with increased neuronal activation in the prefrontal cortex (PFC). Similarly, patients with ASD show enhanced neuronal activation in the PFC in response to tasks involving facial recognition (45) and attention (46). Furthermore, the activation of the dopaminergic system in the PFC of CMA mice was reduced as compared to non-allergic mice (44). Interestingly, in mouse models for ASD as well as in patients with ASD, dampening of the dopaminergic system in the PFC is observed as well (47–49). Taken together, it can be hypothesized that intestinal allergic responses may affect brain circuits involved in social behavior in ASD patients with food allergies.

A variety of studies examined the underlying mechanism of the apparent relation between the induction of food allergy and autistic behavior. As described earlier, individuals with ASD are associated with a pro-inflammatory cytokine profile in various body fluids and a disbalance in Th2 and Treg cells, which are common features for allergic individuals as well (21, 27, 32). Besides differences involving T cells, ASD is also associated with deviations involving the activation of mast cells, which are cells that, upon activation, play a central role in driving allergic reactions. For instance, mast cell activation is shown to be increased in ASD patients, resulting in elevated release of inflammatory and neurotoxic mediators, which in turn could contribute to the development of autistic phenotypes (14, 50, 51). The influence of mast cells on behavior is not limited to the effects of mediator-release, as mast cells can also influence behavior via direct mast cell-neuron interactions, which take place in the brain (52) and the gut (53, 54). The potential of mast cell abnormalities to drive ASD-associated behavior in human is supported by a study performed by Theoharides et al. (55). In this study, the occurrence of mastocytosis, as characterized by the presence of proliferating and hyperactivated mast cells in several organs, is investigated in children with ASD (55). This study indicated that the incidence of mastocytosis is ten times higher in ASD patients than in the general population (55). In addition, 15% of the people who suffered from mastocytosis showed aberrant behaviors, such as concentration problems, decreased attention span, memory impairment, irritation, and distraction (55). These abnormal behaviors are similar to those encountered in ASD, suggesting that hyperactivation of mast cells, as occurs during allergic reactions, might be one of the mechanisms underlying the relation between food allergies and ASD-associated behavior. The potential involvement of mast cell hyperactivation in inducing ASD-associated behavior is further supported by studies showing that mast cell hyperactivation is associated with increased intestinal permeability (55). Accordingly, it is frequently reported that people with ASD have an increased intestinal permeability (56, 57). This disruption of the intestinal barrier might be involved in inducing ASD-like behavior, as preclinical research demonstrated that animals with ASD who suffer from defects in the intestinal barrier show increased levels of bacterial toxins in the bloodstream, which in turn could influence brain function (58).

## The Mammalian Target of Rapamycin in ASD and its Connection With the Immune System

**Figure 2:**
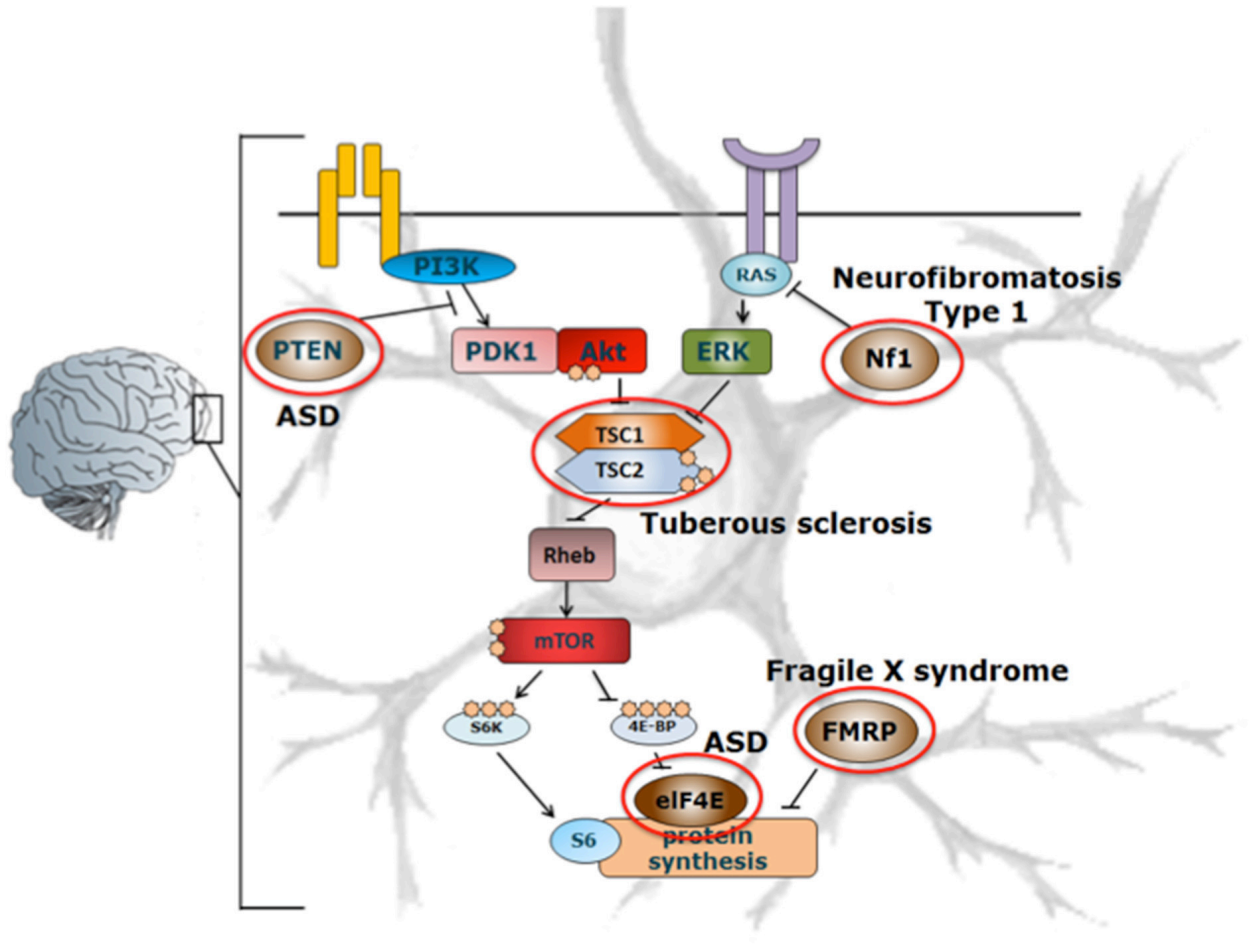
Schematic representation of the mTOR-signaling pathway in ASD. Mutations in various components involved in the mTOR signaling pathway such as PTEN, Nf1, TSC, eIF4E, FMRP (indicated by red circles) affect the protein synthesis machinery, leading to the development of ASD. Adapted from Ehninger et al. (62, 65). With permission of Dr. J. Wu.

ASD is thought to be a neurodevelopmental disorder caused by multiple genetic and environmental factors. The genes involved in ASD are variable and diverse, but mutations in genes related to the mammalian target of rapamycin (mTOR) pathway, including *NF1* (59), *PTEN* (60), *TSC1* (61), *TSC2* (62), *eIF4E* (63), *FMRP* (64), are among those most widely associated with ASD (Figure 2).

**Figure 3:**
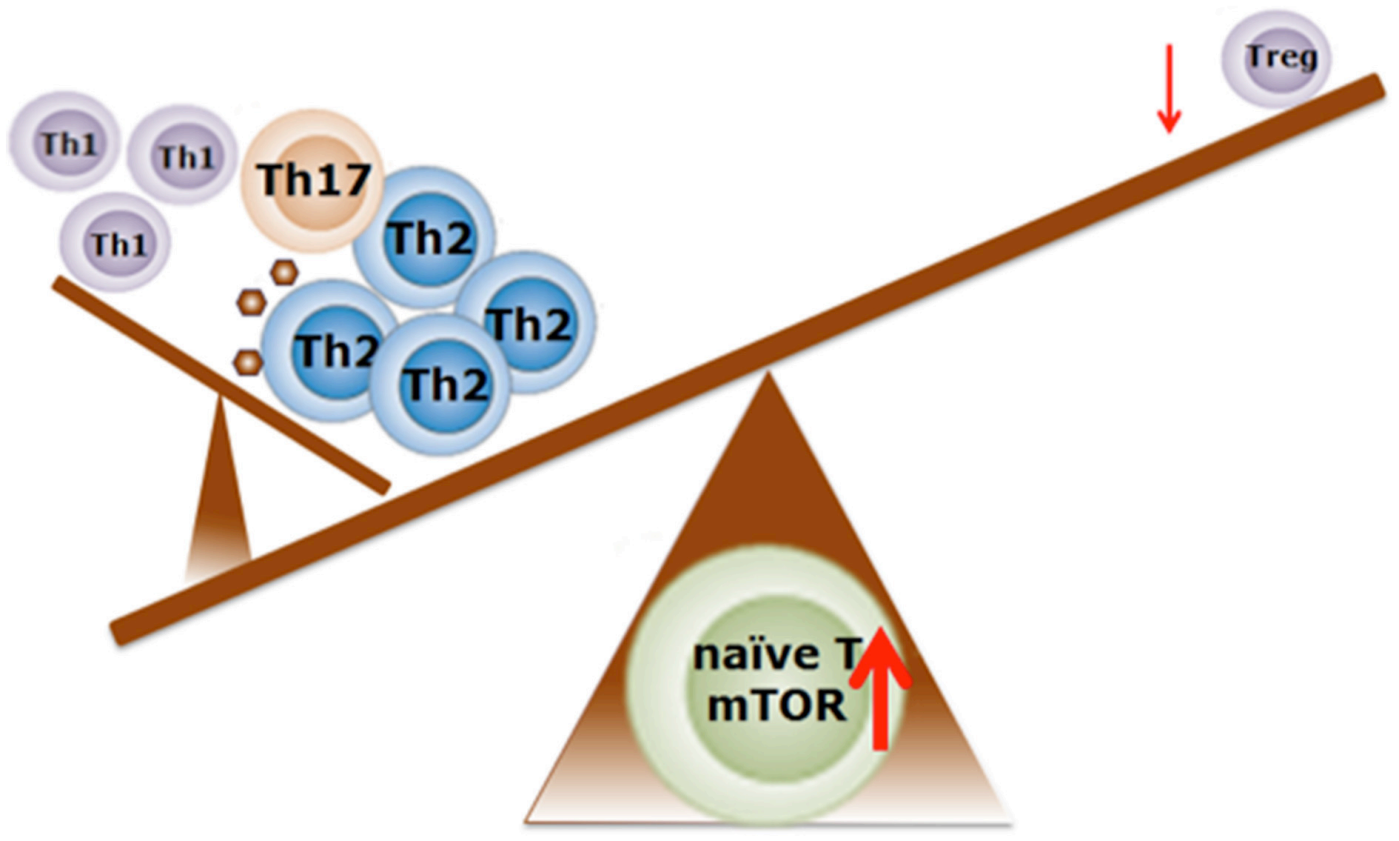
Schematic representation regarding the possible role of mTOR activity in the balance of T cell profiling in food allergy. Enhanced mTOR activity is required for Th1, Th2, and Th17 cell differentiation. Suppression of the mTOR activity induces the differentiation into Treg cells. Adapted from Delgoffe et al. (68) and Kim et al. (69). With permission of Dr J. Wu.

NF1, PTEN, and TSC1/TSC2 act as negative regulators of mTOR complex 1 (mTORC1), of which single gene mutations lead to enhanced mTOR activity in the brain in various mouse models (60, 62, 65). Enhanced activation of mTOR leads to an increase in the phosphorylation of proteins downstream of mTORC1, such as S6 kinase (S6K) and eIF4E-binding proteins (4E-BPs), thereby promoting cap-dependent protein translation (66, 67). In this way, enhanced mTOR activation leads to an increase in the translation of neuroligins (67), which are proteins that are involved in the formation and maintenance of synapses between neurons. In turn, this results in an increased synaptic excitation/inhibition ratio, which may contribute to the development of ASD phenotypes (67). Interestingly, apart from its important role in neurological disorders, the mTOR-signaling pathway is also involved in directing immune responses (Figure 3).

Several studies suggest that Th1 and T helper 17 (Th17) cell differentiation are specifically regulated by mTORC1 signaling, whereas Th2 cell differentiation depends on mTOR complex 2 (mTORC2) signaling (68, 70, 71). This is supported by studies that show that T cells in which mTORC2 activity is eliminated fail to differentiate into Th2 cells *in vitro* and *in vivo*, but are able to differentiate into Th1 and Th17 cells (72). When mTORC1 and mTORC2 are both inhibited using rapamycin, T cells differentiate into Treg cells (72). Indeed, rapamycin-induced mTOR inhibition resulted in elevated numbers of Treg cells in tissue culture of nasal polyps obtained from patients suffering from chronic allergic rhinitis (73). Further evidence of the ability of mTOR to direct immune responses is provided by a study by Kim et al. showing that mTORC1 activation in mast cells is associated with survival, differentiation, migration, and cytokine production (69). Finally, increased mTOR activity is shown to attenuate autophagy (74) which, in the intestine, is an important process to limit chronic intestinal inflammation (75). Thus, hyperactivity of mTOR in the intestinal tract might lead to a loss of immune regulation and barrier integrity, leading to an inflammatory profile possibly associated with allergy. Taken together, these findings reveal that in ASD the mTOR signaling pathways could be disturbed at the level of the immune system, the gastro-intestinal tract as well as the brain. As such, the mTOR signaling pathways could be considered as a central point in the gut-immune-brain axis. Manipulation of the mTOR signaling pathway may therefore serve as a therapeutic strategy for inflammation-associated neurodevelopmental disorders including ASD.

In order to more specifically investigate the role of mTOR in ASD, a variety of mTOR-inhibitors have been used. The mTOR inhibitor that is most commonly used for this purpose is rapamycin. Rapamycin interacts with the intracellular receptor FK506 binding protein (FKBP12). The formed FKBP12-rapapmycin complex binds to mTOR and directly inhibits mTORC1, while mTORC2 is inhibited indirectly in a manner not fully understood (76, 77). As described earlier, mTORC1 drives translation of proteins, like neuroligins, that increase autistic-like phenotypes (77). Therefore, inhibition of mTORC1 by rapamycin might represent a therapeutic strategy for ASD patients. Indeed, rapamycin treatment improved ASD-associated behavior in various animal models for ASD, including the widely used BTBRT(+)ltpr3(tf)/J mouse model (78–81). Moreover, rapamycin treatment was shown to inhibit the social behavioral deficits and normalize the repetitive self-grooming behavior of CMA mice (82). In these mice, the humoral immune responses and mucosal mast cell activation in the intestines were also inhibited (82). At the molecular level, several changes were observed in the intestines of rapamycin-treated CMA mice that might underlie the observed improvement in behavioral outcomes. For instance, it has been shown that rapamycin treatment inhibited the enhanced phosphorylation and activation of the downstream target of mTOR p70 ribosomal protein S6 kinase (p70S6K) in the small intestines (82). Moreover, rapamycin significantly increased the mRNA expression of forkhead box P3 (Foxp3) both in the ileum and the Peyer's Patches of CMA mice, indicating an increase in Treg cell development (82). Finally, rapamycin treatment increased the expression of Treg cell-associated cytokines, including IL-10 and TGF-β in the small intestine of CMA mice (82). Till this day, however, the effects of targeting mTOR on possible immune dysregulations found in the rodent ASD models mentioned above remain understudied.

Collectively, these data support the relevance to further investigate the therapeutic potential of mTOR inhibiting strategies in both genetic and idiopathic forms of ASD. However, the occurrence of serious side effects of chronic administration of pharmacological mTOR inhibitors like rapamycin, such as severe immunosuppression, is reason for concern and limits their potential as a viable treatment option for ASD (83, 84). Hence, safer alternative strategies to normalize mTOR activity are required.

## Targeting ASD-Associated Abnormal mTOR Activity With Amino Acids

Besides pharmacological compounds, nutritional components are also able to modulate mTOR activity. Often described as being the most potent nutrition-derived modulators of mTOR are amino acids (AAs) (85, 86). Although it is not yet fully understood how AAs regulate mTOR activity, AA transporters seem to play a crucial role. A variety of mechanisms involving AA transporters are proposed by which AAs can regulate mTOR activity. First, AA transporters can form a passageway for AAs through the plasma membrane. Once inside the cell, the AAs activate cytoplasmic AA sensors by interacting with a multi-protein complex involving RAG GTPases, Ragulator, and the v-ATPase on the lysosomal surface (87–89). This interaction recruits mTORC1 to lysosomes, where it can be activated. One example of a cell-surface AA transporter is the well-studied heterodimeric CD98, which transports preferably branched chain AAs (BCAAs). In combination with a glutamine transporter (SLC1A5), CD98 exchanges leucine for glutamine to increase the intracellular leucine concentration which, in turn, activates mTORC1 through different signaling molecules (90). Another mechanism by which AAs can modulate mTORC1 activation involves a specific subset of AA transporters, known as AA transceptors, which have a dual transporter-receptor function. The binding or translocation of AAs by transceptors initiates an intracellular signaling cascade, ultimately leading to the modulation of mTORC1 activation (89, 91). Thus, AA transceptors can modulate mTORC1 activation by sensing the extracellular AA concentration. Finally, AAs can also modulate mTORC1 activity by mechanisms that do not involve the translocation of AAs across the plasma membrane (89, 91). Studies have demonstrated that specific AAs can bind and activate G-protein-coupled receptors, which in turn induce lysosomal localization and activation of mTORC1 (89). One example is the G protein-coupled receptor family C group 6 member A (GPRC6A), which is activated by basic, aliphatic and polar AAs such as serine, arginine and lysine.

As AAs can modulate mTORC1 activity via multiple mechanisms, which involve receptors and transceptors that specifically recognize different types of AAs, it is likely that the modulation of mTORC1 activity is different for each type of AA. Consistently, a previous study using mammary epithelial cells demonstrated that the BCAAs leucine (Leu), isoleucine (Ile), and valine (Val) enhanced mTORC1 activity, whereas histidine (His), lysine (Lys), and threonine (Thr) suppressed mTORC1 activity (92). Similar results were found in an *in vitro* allergic mast cell model (93). Inhibition of mTOR signaling by a combination of the AAs His, Lys and Thr in this mast cell model was accompanied by an inhibition of acute mast cell degranulation and an attenuated allergy-associated cytokine production following antigen-IgE mediated activation (93). Based on the outcomes of these *in vitro* studies, a diet containing relatively higher amounts of His, Lys and Thr and relatively lower amounts of Leu, Ile, and Val was developed for preclinical ASD studies (93). It was hypothesized that mTOR signaling could either be normalized or reduced in ASD animals using this AA diet. Consistent with this hypothesis, the AA diet restored the social interaction and normalized the repetitive behavior of CMA mice (93). In addition, dietary intervention with the AAs inhibited the enhanced mTOR signaling pathway in the PFC and amygdala of CMA mice. Since the mTOR signaling pathway is centrally involved in directing immune responses including differentiation of T cells (94), T cell responses were measured as well. Indeed, the CMA-induced Th2 and Th17 cell immune responses were downregulated by the specific AA diet (93). These effects of dietary AAs on effector T cells was accompanied by an upregulation of Treg cells in the small intestines of CMA mice. In another set of experiments using the same AA diet, the diet normalized the repetitive behavior of BTBRT(+)ltpr3(tf)/J mice, a frequently used murine model for ASD (93). This outcome was associated with an inhibition of the enhanced mTOR signaling pathway in the PFC of these mice, suggesting beneficial effects of the AA diet in both genetic and idiopathic forms of ASD (93).

The studies described above further establish mTOR activity to be a crucial factor in the gut-immune-brain axis playing a key role in the regulation of ASD-associated behavior. It has become clear that not only the effects of mTOR on the brain are important, but also the effects of mTOR concerning the regulation of immune responses and intestinal health. This warrants further investigation into the activity status of mTOR in peripheral blood mononuclear cells and intestinal biopsies of autistic patients. These data are needed to further substantiate mTOR as a therapeutic target or as biomarker in patients with ASD. Finally, based on the positive outcomes of an AA-based nutritional intervention on autistic behavior in mouse models for ASD, a proof of principle clinical study in ASD patients using this specific diet is warranted.

## The Effects of Amino Acids on the Gut-Immune-Brain Axis in ASD Beyond mTOR

As described, specific AAs can modulate the activity of mTOR in a manner that might be beneficial for people with ASD. However, the potential of AAs to be beneficial for ASD is not limited to mTOR modulation. Numerous studies have shown that AAs can also exert beneficial effects on other components in the gut-immune-brain axis. Below, the interactions of AAs with the immune system and the gut microbiota (composition) will be discussed in relation to ASD.

## The Potential Effects of Amino Acids on the Derailed Immune System Characteristic for ASD

Beyond targeting the ASD-enhanced mTOR signaling, AAs have been demonstrated to exhibit anti-inflammatory capabilities. As previously described, intestinal and systemic inflammation, as evaluated by a.o. higher levels of pro-inflammatory cytokines (e.g., IL-6, TNFα, and IL1β) are characteristic for people with ASD. In fact, inflammation is shown to induce ASD-associated behavior. Therefore, inhibition of systemic and/or intestinal inflammation might be beneficial for people diagnosed with ASD. Below, the immunomodulatory effects, mostly anti-inflammatory effects, of several AAs are described in relation to ASD.

Numerous studies investigated the immune modulatory functions of AAs in *in vitro* studies. Among these are studies that demonstrate anti-inflammatory effects of AAs on immune cells that might be affected in people with ASD. For instance, application of the BCAAs Leu, Ile and Val individually reduced the production of NO as well as mRNA and protein levels of the pro-inflammatory cytokine IL-6 in LPS-stimulated macrophages *in vitro* (95). Moreover, application of Leu, Ile, and Val individually or in combinations inhibited the acute degranulation and IL-6 production in antigen-IgE-activated mast cells, as previously mentioned (96). Whereas, increasing the availability of BCAAs has anti-inflammatory effects on specified immune cells, a decreased availability of the BCAAs leads to immune cell impairments. For instance, human lymphocytes cultured in medium containing Leu, Ile and Val at levels lower than the typical concentration in adult human plasma showed decreased ability to proliferate (97, 98). Not only *in vitro*, but also *in vivo* experiments demonstrate the importance of an adequate availability of BCAAs for normal immune cell functioning. For example, reducing the levels of Leu, Ile, and Val by 50% in an otherwise normal mice diet increased the susceptibility of mice to salmonella infections, potentially due to an impaired antibody production (99). These mice also showed a lowered number of spleen cells, which might be indicative of a decreased lymphocyte proliferation *in vivo* (99). Interestingly, Novarino et al. identified inactivating mutations in the gene branched chain keto-acid dehydrogenase kinase (BCKDK) in several ASD patients (100). Loss of BCKDK function resulted in hypercatabolism of Leu, Ile and Val, leading to abnormally low levels of these BCAAs in serum and in the brain (100). Knowing the importance of adequate BCAA availability for normal immune cell functioning, these mutations might contribute to the immune impairments observed in a subgroup of individuals with ASD.

In addition to the observed anti-inflammatory effects of the BCAAs *in vitro* and in mouse models, this group of AAs also has demonstrated anti-inflammatory effects in immune compromised humans. For example, in cirrhotic patients, daily supplementation of Leu, Ile and Val improved innate immune cell functions including the phagocytic function of neutrophils and natural killer activity of lymphocytes, which are processes that are also impaired in people with ASD (17, 101, 102). Also, in patients with rectal cancer, infusing a solution enriched in Leu, Ile and Val led to an increase in the number of CD4+ (Th) cells in blood, and led to an increased ratio of CD4+/CD8+ (cytotoxic T) cells in the blood (103). Studies have demonstrated that autistic individuals have fewer CD4+ cells and a lower CD4+/CD8+ cell ratio in blood, indicating immune impairments (104–106). Interestingly, Abd El-Aziz et al. showed a negative correlation between the CD4+/CD8+ cell ratio in blood of individuals with ASD and the severity of autistic behavior. Thus, factors like Leu, Ile and Val that increase the ratio of CD4+/CD8+ cells might provide therapeutic benefits for ASD patients (104).

Besides their modulatory capacities on peripheral immune cells, the BCAAs also affect microglial cells, the immune cells of the brain. Culturing rat microglial cells in a medium enriched with these BCAAs induced a more anti-inflammatory state of the microglial cells after LPS stimulation (107). This was supported by a decreased microglial production of IL-1β, TNFα, and inducible nitric-oxide synthase (iNOS), as well as an increased production of IL-10, both at the level of mRNA and at the protein level (107). Moreover, microglial phagocytic capacity, which is critical for normal brain functioning and is impaired in individuals with ASD (108), was stimulated by the presence of high levels of the BCAAs (107). However, neuroprotective functions of microglial cells were impaired in the presence of high levels of Leu, Ile and Val, as represented by a decreased production of the neuroprotective factor Insulin-like growth factor 1 (IGF-1) (107). The latter observation is in line with the possible detrimental effects of mTOR-hyperactivation.

Alongside the BCAAs, the essential AAs Thr, Lys, and His also have immunomodulating capacities, potentially via modulation of mTOR signaling (93). Of these AAs, Thr has been most widely associated with immunomodulation *in vivo*. Animal studies have demonstrated that Thr is critical for intestinal barrier and immune function and can improve intestinal epithelial integrity in inflammatory conditions where the gut barrier is impaired, as is the case in ASD (109). For instance, in various rat models of colitis, a Thr-enriched diet was shown to restore mucus synthesis and normalize the gut microbiota, thereby improving intestinal function (110, 111). Studies in animals and humans also suggest an increased requirement of Thr by the intestines in inflammatory conditions in order to maintain intestinal barrier function (112, 113). Although less widely studied, the AA His also interacts with the immune system. Apart from its use for the generation of histamine, a key molecule involved in immune responses, His exhibits anti-inflammatory effects on a variety of immune cells. For instance, *in vitro* studies show that supplementation of His suppressed IL-8 secretion in TNFα-activated human epithelial cells, PBMCs and monocytes (114, 115). The immunomodulating ability of His is also demonstrated *in vivo*, as dietary His improved murine colitis by inhibiting the production of pro-inflammatory cytokines by macrophages (116). Studies examining the immunomodulatory effects of Lys are limited. However, dietary deficiency of Lys has been shown to limit lymphocyte proliferation and induces a pro-inflammatory state, represented by increased levels of pro-inflammatory cytokines and NO in the kidney, liver and spleen of piglets (117). Taken together, data from both *in vitro* and *in vivo* experiments suggest that there is a potential for the AAs Thr, Lys, and His to ameliorate the compromised gut-immune-brain axis in ASD.

In addition to the AAs that are demonstrated to interact with mTOR, several other AAs also have anti-inflammatory capabilities, in particular glycine (Gly), glutamine (Gln) and glutamate (Glu). *In vitro* studies demonstrated that supplementation of Gly protects intestinal epithelial cells against inflammatory oxidative stress (118), and reduces pro-inflammatory cytokine production while enhancing IL-10 expression of LPS-stimulated monocytes (119). The capacity of Gly to exert anti-inflammatory effects is further supported by *in vivo* studies using a variety of animal immune models. For instance, oral intake of Gly reduced TNFα production and improved the survival rate of LPS-injected rats (120), reduced inflammation and oxidative stress in a mouse model of cancer cachexia (121) and inhibited the onset of CMA in mice (122). As the pro-inflammatory, allergic-like status of the intestines in people with ASD might play a role in the induction of autistic-like behavior, it can be speculated that dietary Gly supplementation might have therapeutic potential for individuals with ASD.

The AAs Gln and Glu are related to each other, both in terms of structure and their immunomodulatory capabilities. Both Gln and Glu are critical for intestinal growth and functions and are shown to reduce intestinal hyperpermeability both *in vitro* and *in vivo* (123–125). Remarkably, plasma levels of Gln are reported to be lower in patients with ASD as compared to healthy individuals (126). The reduced bioavailability of Gln might be involved in the mucosal barrier impairments observed in individuals with ASD. This is supported by studies demonstrating that low levels of serum Gln correlate with intestinal barrier disruption and inflammation in children (127, 128). Besides its involvement in intestinal barrier function, Gln is also widely known as an anti-inflammatory agent, likely via inhibition of the activation of nuclear factor kappa B (NF-κB) and signal transducers and activators of transcription (STAT) (129). In turn, this inhibition leads to suppression of the production of inflammatory cytokines such as IL-6, TNFα and IL-8 as shown for various immune cells *in vitro*, in animal immune models and in immune-compromised human (129–131). As such, Gln is proposed as candidate for treating inflammatory disorders. Anti-inflammatory effects of Glu include the inhibition of Th2 cells, the stimulation of the production of regulatory cytokine IL-10 by Treg cells and the stimulation of Treg cell development via modulation of the cytokine expression by dendritic cells (132, 133).

Besides their involvement in inflammatory immune responses, Gly and Glu are also neuroactive AAs. Glu is the primary excitatory neurotransmitter of the brain, which is stored in the form of Gln until it is transferred to presynaptic terminals and converted back to Glu (134). Normally, Glu has a protective effect on cognitive function and neural plasticity. However, excessive levels of Glu may be neurotoxic and may play a role in neuroinflammation events in the pathogenesis of ASD (135). Accordingly, multiple studies report higher levels of Glu in serum, plasma and the brain of people with ASD as compared to healthy controls (136–138). In contrast, levels of Gly, a major inhibitory neurotransmitter, are found to be unchanged in patients with ASD (126). The imbalance between Gly and Gln might drive an imbalance in inhibitory and excitatory neurotransmitters, which is indicated to lead to excessive neuroinflammation (137).

As discussed, specific AAs can modify the functions of the immune system in a way that might be beneficial for individuals with ASD. Conversely, the derailed immune system observed in ASD can also modulate the bioavailability of specific AAs, and thereby drive the pathogenesis of ASD. This has been demonstrated most clearly for tryptophan (Trp), of which plasma levels are consistently shown to be lower in ASD patients (139–141). Apart from being a precursor for the neurotransmitter serotonin, Trp is extensively metabolized in the kynurenine pathway (142). This pathway produces several immunomodulatory and neuroactive compounds, examples of which include kynurenine (142) and quinolinic acid (143), respectively. In pro-inflammatory conditions, like ASD, the activation of the kynurenine pathway is enhanced (144), leading to abnormal production of metabolites with neurotoxic effects (144, 145). Furthermore, the kynurenine pathway produces metabolites that induce apoptosis in Th1 cells but not in Th2 cells, suggesting that an increased activation of this pathway favors Th2 polarization, which is characteristic for ASD (146). Finally, aberrant activation of the kynurenine pathway in ASD also diverts Trp from the serotonin synthesis route (144, 147, 148). Psychological complications characteristic for a.o. ASD patients may originate from serotonin deficiency mediated by the depletion of Trp following kynurenine pathway activation (139, 148), which is also reported in a murine model for ASD (149, 150). Whereas, aberrant Trp metabolism might induce neurotoxicity and a Th2-type immune status, Trp can also act as an anti-inflammatory agent. For instance, dietary supplementation of Trp in a mouse and piglet model for colitis reduced inflammation (151, 152), whereas mice fed a low-Trp diet became increasingly susceptible to chemically induced inflammation (153). Promising effects of a multi-nutrient diet containing high Trp on disturbed behavior and reduced intestinal and CNS serotonin levels are reported in a murine model for ASD (96). Whether dietary supplementation of Trp has therapeutic potential for ASD patients remains open to speculation: it might further drive abnormal activation of the kynurenine pathway, however, it could also provide benefits by reducing inflammation and normalizing serotonin synthesis.

## The Interaction Between Amino Acids and the Gut Microbiome in Relation to ASD

Accumulating evidence has demonstrated a bidirectional communication between the brain and the gut, with the intestinal microbiota as a key factor in both health and disease (154, 155). The microbiota can affect cognition and behavior, as has been demonstrated in an increasing number of studies (156–158). Modification of the microbiota composition or absence of the microbiota impacts the hypothalamic-pituitary-adrenal (HPA) axis, the immune system and behavior (158). The importance of gut microbiota in early life and brain development is demonstrated by animal studies showing that maternal separation of neonates does not only lead to a dysbiotic state of the microbiota that persists into adulthood, but also results in long-term cognitive and behavioral deficits (159). Nevertheless, the mechanistic link between the gut microbiota, gut immune homeostasis, and brain functioning remains poorly understood. A variety of mechanisms including immune, hormonal and neural pathways are likely to be involved and may depend on the nature of the microbiota dysbiosis. Also for ASD, the microbiome-gut-immune-brain axis is suggested to be important as profound changes in the microbial composition and activity have been reported (160). Below, we describe the possible interaction between AAs and the gut microbiota in perspective of ASD.

Over the last decades extensive studies have revealed that the gut microbiota composition plays an important role in the metabolism and recycling of nitrogen-containing compounds (161, 162). One of the major nitrogenous compounds utilized by gut bacteria are AAs. AAs derived from either the host or from food enter the gut and are metabolized by gut bacteria into a wide variety of products (163). Hence, it can be speculated that the gut microbiota regulates the AA pool and composition that is available to the host. This is supported by a study demonstrating that gut bacteria alter the AA distribution in the gastrointestinal tract (164). Alterations in AA availability may lead to changes in signaling pathways that are sensitive to AAs, including the mTOR signaling and inflammatory pathways (77, 165). For instance, studies demonstrated that people with ASD have lower BCAA levels in their plasma, urine and cerebrospinal fluid (141, 166, 167). A deficiency of any of the BCAAs is shown to cause immune cell impairments in various animal models, leading to a more pro-inflammatory immune status (168), which is also observed in people with ASD. A possible explanation for the abnormal levels of BCAAs might be an increased conversion of these AAs by gut *Proteobacteria*, as these are among the most extensive BCAA-fermenting bacteria (169) and are shown to be more abundant in people with ASD (170–172).

Gut bacteria do not only alter AA availability, but they also play a role in the availability of AA-derived metabolites. For instance, levels of Thr are shown to be decreased in patients with ASD (141, 167, 173). Gut bacteria use Thr for a.o. the production of short-chain fatty acids (SCFAs), mainly propionate (163, 174). Studies have revealed an increased abundance of propionate in people with ASD (175, 176). In fact, elevated propionate production is postulated to be involved in the pathogenesis of ASD, as this SCFA is shown to cross the blood-brain barrier and is able to induce ASD-like behavior in adult rodents, possibly via activation of microglia (177–179). The gut microbiome also has a role in the regulation Trp metabolism (180, 181). Deviations in either endogenous or bacterial Trp metabolism have been associated with a variety of immune-related disorders, including ASD (182, 183). For instance, a recent study demonstrated a significant reduction of serotonin bioavailability in the intestines of a mouse models for ASD (96, 149). This reduction was accompanied by a down-regulation of the gene *Tph1*, indicating a lower production of serotonin from dietary Trp (149). Interestingly, serotonin production from Trp was positively associated with the abundance of the *Blautia* bacteria present in the gut. In both mouse models of ASD and in humans with ASD, the abundance of *Blautia* bacteria is reported to be lower compared to healthy controls (149, 184). This change in the microbiota might underlie the abnormal Trp-metabolism and serotonin bioavailability that is frequently observed in ASD patients as well as in *in vivo* models for ASD and thus may drive ASD pathogenesis.

As discussed, the gut microbiota influences the bioavailability of AAs and their metabolites. Vice versa, the availability of AAs can also influence the microbiota composition. For instance, several studies have reported that the gut microbiota of people with ASD as well as in murine ASD models is characterized by a significantly higher ratio of *Firmicutes* to *Bacteroidetes*, mainly due to a decrease in *Bacteroidetes* (171, 185, 186). The ratio of *Firmicutes* to *Bacteroidetes*, which are the two most abundant bacterial phyla in human, is considered of significant relevance in human health (187). An increased *Firmicutes/Bacteroidetes* ratio might be involved in inducing inflammation, as this is observed in several inflammatory conditions and as this ratio is shown to be positively associated with dysregulated humoral and cellular immunity (188, 189). Interestingly, dietary supplementation of arginine (Arg) in healthy mice decreased the *Firmicutes* to *Bacteroidetes* ratio in the small intestine, mainly due to an increase in *Bacteroidetes* (190). Similarly, dietary supplementation of Gln decreased the *Firmicutes/Bacteroidetes* ratio in sows (191). Whether modulation of Gln and Arg intake can restore the aberrant *Firmicutes/Bacteroidetes* ratio observed in ASD patients, and subsequently improve their immune status remains to be examined.

Furthermore, several studies have reported an increase in the abundance of *Proteobacteria* in people with ASD (170–172). *Proteobacteria* are often found to be increased in inflammatory conditions such as colitis and inflammatory bowel disease and have been identified as a marker for microbiota instability and gut inflammation (192, 193). Supplementation of BCAAs was shown to largely reduce the abundance of *Proteobacteria* in mice, accompanied by lower concentrations of inflammatory marker LPS-binding protein (LBP) in serum (194), which has been shown to be elevated in ASD patients (195). In addition, BCAA supplementation also reduced the *Firmicutes/Bacteroidetes* ratio in middle-aged mice (194). However, whether BCAA supplementation is able to restore the abnormal gut microbiota in ASD, and which underlying mechanisms are involved remains to be further investigated.

## Conclusion

**Figure 4:**
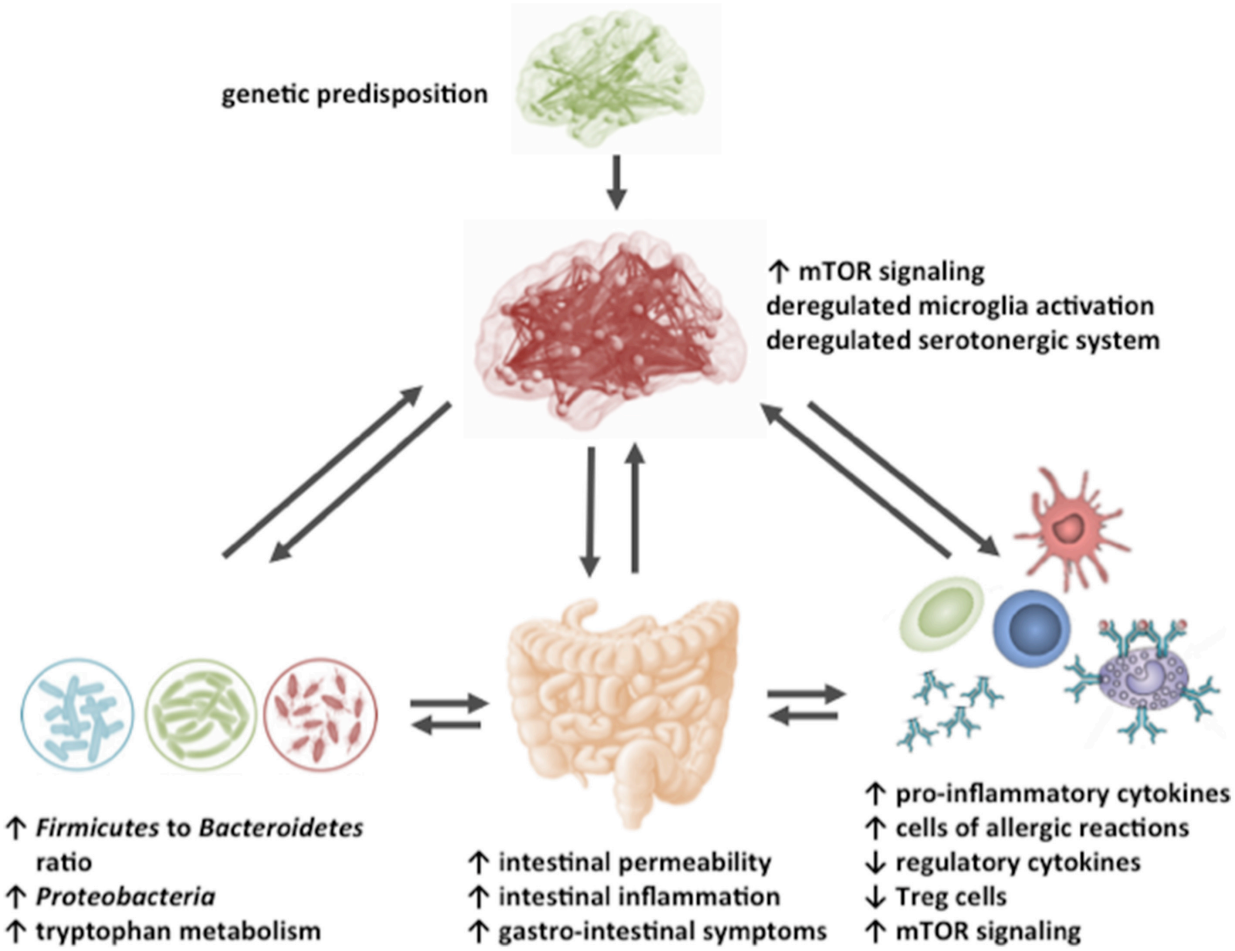
Schematic representation of the possible targets for dietary amino acids in the impaired gut-microbiome-immune-brain axis in ASD.

In Table 1, an overview is presented of the complex roles of AAs described in this review regarding the microbiome-gut-immune-brain axis in ASD. Besides behavioral deficits, people with ASD are characterized by systemic inflammation, gastrointestinal immune-related disturbances and changes in the gut microbiota composition. Moreover, differences in levels of specific AAs in various body compartments, including the intestinal tract, blood, urine and brain have been reported in patients with ASD, as well as in rodent models for ASD. This review described that specific AAs can modulate the intestinal epithelial immune barrier and are able to tune the mucosal immune system, possibly through influencing the mTOR pathway in immune cells. Moreover, specific AAs can influence neuroinflammatory processes and can target the aberrant mTOR signaling in the brain, thereby influencing neuronal activity and disturbed behavior associated with ASD. Finally, though limited reports are available, dietary AAs can influence the changed intestinal microbiota composition and activity of people with ASD. Figure 4 shows a graphical overview of the different targets for dietary AAs in ASD. Taken together, this review creates more insight in the complex pathogenic relevance of the microbiome-gut-immune-brain axis in ASD and warrants additional research into the opportunities for AA-based nutritional interventions as treatment for ASD in the system medicine approach.

**Table 1:** Overview of the complex roles of amino acids in the microbiome-gut-immune-brain axis in ASD.

**AA**	**Body fluid levels in ASD**	**Effects on the microbiota**		**Effects on the intestines**		**Effects on the immune system**				**Effects on mTOR**	**Effects in the brain**	
Leu	**↓** Blood, urine, CSF (126)	**↓** Proteobacteria (194)	**↓** F/B (194)			**↓** MC activation (93)	**↓** Inflammatory response mϕ (95)	Essential for lymphocyte proliferation (97, 98)	Essential for Ig production (100)	**↑** MC and EC (92)	**↑** Microglia phagocytosis (107)	**↓** Neuro-protective factor microglia (107)
Ile	**↓** Blood, urine, CSF (126)	**↓** Proteobacteria (194)	**↓** F/B (194)			**↓** MC activation (93)	**↓** Inflammatory response mϕ (95)	Essential for lymphocyte proliferation (97, 98)	Essential for Ig production (100)	**↑** MC and EC (92)	**↑** Microglia phagocytosis (107)	**↓** Neuro-protective factor microglia (107)
Val	**↓** Blood, urine, CSF (126)	**↓** Proteobacteria (194)	**↓** F/B (194)			**↓** MC activation (93)	**↓** Inflammatory response mϕ (95)	Essential for lymphocyte proliferation (97, 98)	Essential for Ig production (100)	**↑** MC and EC (92)	**↑** Microglia phagocytosis (107)	**↓** Neuro-protective factor microglia (107)
His	**↑** Blood (126) **↓** Urine (126)			**↓** Inflammatory response EC (115)		**↓** MC activation (93)	**↓** Th2 and Th17 cells (93)	**↑** Treg cells (93)	**↓** Inflammatory response mϕ (116) and PBMC (114, 115)	**↓** Brain, MC and EC (92)		
Thr	**↓** Blood (126)	Fermented into SCFA (163, 174)		**↑** Epithelial barrier (109)	**↑** Mucus production (110, 111)	**↓** MC activation (93)	**↓** Th2 and Th17 cells (93)	**↑** Treg cells (93)		**↓** Brain, MC and EC (92)		
Lys	**↑** Blood, Urine (126)					**↓** MC activation (93)	**↓** Th2 and Th17 cells (93)	**↑** Treg cells (93)	Essential for downregulating inflammatory cytokines and NO production (117)	**↓** Brain, MC and EC (92)		
Gly	- (126)			Protects EC (118)	**↓** Intestinal inflammation (120–122)	**↓** Inflammatory response mϕ (119)						
Gln	**↓** Blood (126)	**↓** F/B (191)		**↑** Epithelial barrier (123)		**↓** MC activation (131)	**↓** Inflammatory cytokine and iNOS production in various immune models (129–131)					
Glu	**↑** Blood and brain (126)			**↑** Epithelial barrier (124, 125)		**↓** Th2 cells (132, 133)	**↑** Treg cells and regulatory cytokine IL-10 (132, 133)				Neurotoxic at high levels (135)	
Arg	- (126)	**↓** F/B (190)										
Trp	**↓** Blood (126)			**↓** Intestinal inflammation (151, 152)	Essential for serotonin synthesis (149, 150)							Essential for serotonin synthesis (149, 150)

*A selection of the complex effects of AAs on (from left to right) the microbiota composition, intestinal inflammation and intestinal barrier function, the immune system, mTOR activity and on cells in the brain. This table is limited to the effects that are relevant for ASD. The **↓** indicates a downregulation of the corresponding target by the specific amino acid, whereas the **↑** indicates an upregulation. Leu, leucine; Ile, isoleucine; Val, valine; His, histidine; Thr, threonine; Lys, lysine; Gly, glycine; Gln, glutamine; Glu, glutamate; Arg, arginine; Trp, tryptophan; MC, mast cell; EC, epithelial cell; CSF, cerebrospinal fluid; SCFA, short chain fatty acids; F/B, Firmicutes/Bacteroidetes ratio; PBMC, peripheral blood mononuclear cells; Ig, immunoglobulin; LPS, lipopolysaccharide; NO, nitric oxide; iNOS, inducible nitric oxide synthase; mΦ, macrophages; Th2, T helper 2 cells; Th17: T helper 17 cells; Treg, regulatory T cells*.

## Author Contributions

All authors listed have made a substantial, direct and intellectual contribution to the work, and approved it for publication.

### Conflict of Interest Statement

JvS, JG, JvB, AH are current employees of Danone Nutricia Research. The remaining authors declare that the research was conducted in the absence of any commercial or financial relationships that could be construed as a potential conflict of interest.

## References

[1] Tchaconas A Adesman A. Autism spectrum disorders: a pediatric overview and update. Curr Opin Pediatr. (2013) 25:130–44. 10.1097/MOP.0b013e32835c2b70 23274432

[2] Williams JG Higgins JP Brayne CE. Systematic review of prevalence studies of autism spectrum disorders. Arch Dis Child. (2006) 91:8–15. 10.1136/adc.2004.062083 15863467 PMC2083083

[3] Fombonne E. Epidemiology of pervasive developmental disorders. Pediatr Res. (2009) 65:591–8. 10.1203/PDR.0b013e31819e7203 19218885

[4] Elsabbagh M Divan G Koh YJ Kim YS Kauchali S Marcín C. Global prevalence of autism and other pervasive developmental disorders. Autism Res. (2012) 5:160–79. 10.1002/aur.239 22495912 PMC3763210

[5] Hansen SN Schendel DE Parner ET. Explaining the increase in the prevalence of autism spectrum disorders: the proportion attributable to changes in reporting practices. JAMA Pediatr. (2015) 169:56–62. 10.1001/jamapediatrics.2014.1893 25365033

[6] Kim YS Leventhal BL Koh YJ Fombonne E Laska E Lim EC. Prevalence of autism spectrum disorders in a total population sample. Am J Psychiatry. (2011) 168:904–12. 10.1176/appi.ajp.2011.10101532 21558103

[7] Lyall K Croen L Daniels J Fallin MD Ladd-Acosta C Lee BK. The changing epidemiology of autism spectrum disorders. Annu Rev Public Health. (2017) 38:81–102. 10.1146/annurev-publhealth-031816-044318 28068486 PMC6566093

[8] Minakova E Warner BB. Maternal immune activation, central nervous system development and behavioral phenotypes. Birth Defects Res. (2018) 110:1539–50. 10.1002/bdr2.1416 30430765

[9] Vuong HE Hsiao EY. Emerging roles for the gut microbiome in autism spectrum disorder. Biol Psychiatry. (2017) 81:411–23. 10.1016/j.biopsych.2016.08.024 27773355 PMC5285286

[10] Bauman ML. Medical comorbidities in autism: challenges to diagnosis and treatment. Neurotherapeutics. (2010) 7:320–7. 10.1016/j.nurt.2010.06.001 20643385 PMC5084236

[11] Onore C Careaga M Ashwood P. The role of immune dysfunction in the pathophysiology of autism. Brain Behav Immun. (2012) 26:383–92. 10.1016/j.bbi.2011.08.007 21906670 PMC3418145

[12] McElhanon BO McCracken C Karpen S Sharp WG. Gastrointestinal symptoms in autism spectrum disorder: a meta-analysis. Pediatrics. (2014) 133:872–83. 10.1542/peds.2013-3995 24777214

[13] Careaga M Van de Water J Ashwood P. Immune dysfunction in autism: a pathway to treatment. Neurotherapeutics. (2010) 7:283–92. 10.1016/j.nurt.2010.05.003 20643381 PMC5084232

[14] Theoharides TC. Is a subtype of autism an allergy of the brain? Clin Ther. (2013) 35:584–91. 10.1016/j.clinthera.2013.04.009 23688533

[15] Edmiston E Ashwood P Van de Water J. Autoimmunity, autoantibodies, and autism spectrum disorder. Biol Psychiatry. (2017) 81:383–90. 10.1016/j.biopsych.2016.08.031 28340985 PMC5373490

[16] Estes ML McAllister AK. Immune mediators in the brain and peripheral tissues in autism spectrum disorder. Nat Rev Neurosci. (2015) 16:469–86. 10.1038/nrn3978 26189694 PMC5650494

[17] Meltzer A Van de Water J. The role of the immune system in autism spectrum disorder. Neuropsychopharmacology. (2017) 42:284–98. 10.1038/npp.2016.158 27534269 PMC5143489

[18] Morgan JT Chana G Pardo CA Achim C Semendeferi K Buckwalter J. Microglial activation and increased microglial density observed in the dorsolateral prefrontal cortex in autism. Biol Psychiatry. (2010) 68:368–76. 10.1016/j.biopsych.2010.05.024 20674603

[19] Vargas DL Nascimbene C Krishnan C Zimmerman AW Pardo CA. Neuroglial activation and neuroinflammation in the brain of patients with autism. Ann Neurol. (2005) 57:67–81. 10.1002/ana.20315 15546155

[20] Atladottir HO Pedersen MG Thorsen P Mortensen PB Deleuran B Eaton WW. Association of family history of autoimmune diseases and autism spectrum disorders. Pediatrics. (2009) 124:687–94. 10.1542/peds.2008-2445 19581261

[21] Molloy CA Morrow AL Meinzen-Derr J Schleifer K Dienger K Manning-Courtney P. Elevated cytokine levels in children with autism spectrum disorder. J Neuroimmunol. (2006) 172:198–205. 10.1016/j.jneuroim.2005.11.007 16360218

[22] Lee LC Zachary AA Leffell MS Newschaffer CJ Matteson KJ Tyler JD. Hla-dr4 in families with autism. Pediatr Neurol.. (2006) 35:303–7. 10.1016/j.pediatrneurol.2006.06.006 17074598

[23] Odell D Maciulis A Cutler A Warren L McMahon WM Coon H. Confirmation of the association of the c4b null allelle in autism. Hum Immunol. (2005) 66:140–5. 10.1016/j.humimm.2004.11.002 15694999

[24] Ashwood P Enstrom A Krakowiak P Hertz-Picciotto I Hansen RL Croen LA. Decreased transforming growth factor beta1 in autism: a potential link between immune dysregulation and impairment in clinical behavioral outcomes. J Neuroimmunol. (2008) 204:149–53. 10.1016/j.jneuroim.2008.07.006 18762342 PMC2615583

[25] Enstrom A Onore C Hertz-Picciotto I Hansen R Croen L Van de Water J. Detection of il-17 and il-23 in plasma samples of children with autism. Am J Biochem Biotechnol. (2008) 4:114–20. 10.3844/ajbbsp.2008.114.120 27688738 PMC5038352

[26] Okada K Hashimoto K Iwata Y Nakamura K Tsujii M Tsuchiya KJ. Decreased serum levels of transforming growth factor-beta1 in patients with autism. Prog Neuropsychopharmacol Biol Psychiatry. (2007) 31:187–90. 10.1016/j.pnpbp.2006.08.020 17030376

[27] Hashim H Abdelrahman H Mohammed D Karam R Association between plasma levels of transforming growth factor-β1, il-23 and il-17 and the severity of autism in egyptian children. Res Autism Spectr Disord. (2013) 7:199–204. 10.1016/j.rasd.2012.08.007

[28] Mostafa GA Al Shehab A Fouad NR. Frequency of cd4+cd25high regulatory t cells in the peripheral blood of egyptian children with autism. J Child Neurol. (2010) 25:328–35. 10.1177/0883073809339393 19713552

[29] Shevach EM Tran DQ Davidson TS Andersson J. The critical contribution of tgf-beta to the induction of foxp3 expression and regulatory t cell function. Eur J Immunol. (2008) 38:915–7. 10.1002/eji.200738111 18395859 PMC2662375

[30] Gomes FC Sousa Vde O Romao L. Emerging roles for tgf-beta1 in nervous system development. Int J Dev Neurosci. (2005) 23:413–24. 10.1016/j.ijdevneu.2005.04.001 15936920

[31] Sanyal S Kim SM Ramaswami M. Retrograde regulation in the cns; neuron-specific interpretations of tgf-beta signaling. Neuron. (2004) 41:845–8. 10.1016/S0896-6273(04)00152-7 15046717

[32] Ashwood P Krakowiak P Hertz-Picciotto I Hansen R Pessah I Van de Water J. Elevated plasma cytokines in autism spectrum disorders provide evidence of immune dysfunction and are associated with impaired behavioral outcome. Brain Behav Immun. (2011) 25:40–5. 10.1016/j.bbi.2010.08.003 20705131 PMC2991432

[33] Ashwood P Krakowiak P Hertz-Picciotto I Hansen R Pessah IN Van de Water J. Associations of impaired behaviors with elevated plasma chemokines in autism spectrum disorders. J Neuroimmunol. (2011) 232:196–9. 10.1016/j.jneuroim.2010.10.025 21095018 PMC3053074

[34] Leigh LY Spergel JM An in-depth characterization of a large cohort of adults patients with eosinophilic esophagitis. Ann Allergy Asthma Immunol. (2018) 122:65–72.e1. 10.1016/j.anai.2018.09.452 30223114

[35] Theoharides TC Tsilioni I Patel AB Doyle R. Atopic diseases and inflammation of the brain in the pathogenesis of autism spectrum disorders. Transl Psychiatry. (2016) 6:e844. 10.1038/tp.2016.77 27351598 PMC4931610

[36] NIAID-Sponsored Expert Panel Boyce JA Assa'ad A Burks AW Jones SM Sampson HA Guidelines for the diagnosis and management of food allergy in the united states: report of the niaid-sponsored expert panel. J Allergy Clin Immunol. (2010) 126:S1–58. 10.1016/j.jaci.2010.10.007 21134576 PMC4241964

[37] Kobayashi T Iijima K Dent AL Kita H. Follicular helper t cells mediate ige antibody response to airborne allergens. J Allergy Clin Immunol. (2017) 139:300–313.e307. 10.1016/j.jaci.2016.04.021 27325434 PMC5115999

[38] Ho MH Wong WH Chang C. Clinical spectrum of food allergies: a comprehensive review. Clin Rev Allergy Immunol. (2014) 46:225–40. 10.1007/s12016-012-8339-6 23229594

[39] de Theije CG Wu J da Silva SL Kamphuis PJ Garssen J Korte SM Pathways underlying the gut-to-brain connection in autism spectrum disorders as future targets for disease management. Eur J Pharmacol. (2011) 668 (Suppl. 1):S70–80. 10.1016/j.ejphar.2011.07.013 21810417

[40] Gurney JG McPheeters ML Davis MM. Parental report of health conditions and health care use among children with and without autism: National survey of children's health. Arch Pediatr Adolesc Med. (2006) 160:825–30. 10.1001/archpedi.160.8.825 16894082

[41] Lyall K Van de Water J Ashwood P Hertz-Picciotto I. Asthma and allergies in children with autism spectrum disorders: Results from the charge study. Autism Res. (2015) 8:567–74. 10.1002/aur.1471 25722050 PMC6900397

[42] Sanctuary MR Kain JN Angkustsiri K German JB. Dietary considerations in autism spectrum disorders: the potential role of protein digestion and microbial putrefaction in the gut-brain axis. Front Nutr. (2018) 5:40. 10.3389/fnut.2018.00040 29868601 PMC5968124

[43] Lucarelli S Frediani T Zingoni AM Ferruzzi F Giardini O Quintieri F. Food allergy and infantile autism. Panminerva Med. (1995) 37:137–41. 8869369

[44] de Theije CG Wu J Koelink PJ Korte-Bouws GA Borre Y Kas MJ Autistic-like behavioural and neurochemical changes in a mouse model of food allergy. Behav Brain Res. (2014) 261:265–74. 10.1016/j.bbr.2013.12.008 24333575

[45] Dalton KM Nacewicz BM Johnstone T Schaefer HS Gernsbacher MA Goldsmith HH. Gaze fixation and the neural circuitry of face processing in autism. Nat Neurosci. (2005) 8:519–26. 10.1038/nn1421 15750588 PMC4337787

[46] Belmonte MK Gomot M Baron-Cohen S. Visual attention in autism families: 'Unaffected' sibs share atypical frontal activation. J Child Psychol Psychiatry. (2010) 51:259–76. 10.1111/j.1469-7610.2009.02153.x 19912448

[47] Hara Y Takuma K Takano E Katashiba K Taruta A Higashino K. Reduced prefrontal dopaminergic activity in valproic acid-treated mouse autism model. Behav Brain Res. (2015) 289:39–47. 10.1016/j.bbr.2015.04.022 25907743

[48] Paul K Venkitaramani DV Cox CL. Dampened dopamine-mediated neuromodulation in prefrontal cortex of fragile x mice. J Physiol. (2013) 591:1133–43. 10.1113/jphysiol.2012.241067 23148316 PMC3591719

[49] Ernst M Zametkin AJ Matochik JA Pascualvaca D Cohen RM. Low medial prefrontal dopaminergic activity in autistic children. Lancet. (1997) 350:638. 10.1016/S0140-6736(05)63326-0 9288051

[50] Theoharides TC Angelidou A Alysandratos KD Zhang B Asadi S Francis K. Mast cell activation and autism. Biochim Biophys Acta. (2012) 1822:34–41. 10.1016/j.bbadis.2010.12.017 21193035

[51] Theoharides TC Stewart JM Panagiotidou S Melamed I. Mast cells, brain inflammation and autism. Eur J Pharmacol. (2016) 778:96–102. 10.1016/j.ejphar.2015.03.086 25941080

[52] Rozniecki JJ Dimitriadou V Lambracht-Hall M Pang X Theoharides TC. Morphological and functional demonstration of rat dura mater mast cell-neuron interactions *in vitro* and *in vivo*. Brain Res. (1999) 849:1–15. 10.1016/S0006-8993(99)01855-7 10592282

[53] Buhner S Schemann M. Mast cell-nerve axis with a focus on the human gut. Biochim Biophys Acta. (2012) 1822:85–92. 10.1016/j.bbadis.2011.06.004 21704703

[54] Schemann M Camilleri M. Functions and imaging of mast cell and neural axis of the gut. Gastroenterology. (2013) 144:698–704.e694. 10.1053/j.gastro.2013.01.040 23354018 PMC3922647

[55] Theoharides TC. Autism spectrum disorders and mastocytosis. Int J Immunopathol Pharmacol. (2009) 22:859–65. 10.1177/039463200902200401 20074449

[56] de Magistris L Familiari V Pascotto A Sapone A Frolli A Iardino P Alterations of the intestinal barrier in patients with autism spectrum disorders and in their first-degree relatives. J Pediatr Gastroenterol Nutr. (2010) 51:418–24. 10.1097/MPG.0b013e3181dcc4a5 20683204

[57] Konig J Wells J Cani PD Garcia-Rodenas CL MacDonald T Mercenier A. Human intestinal barrier function in health and disease. Clin Transl Gastroenterol. (2016) 7:e196. 10.1038/ctg.2016.54 27763627 PMC5288588

[58] Hsiao EY McBride SW Hsien S Sharon G Hyde ER McCue T. Microbiota modulate behavioral and physiological abnormalities associated with neurodevelopmental disorders. Cell. (2013) 155:1451–63. 10.1016/j.cell.2013.11.024 24315484 PMC3897394

[59] Costa RM Federov NB Kogan JH Murphy GG Stern J Ohno M. Mechanism for the learning deficits in a mouse model of neurofibromatosis type 1. Nature. (2002) 415:526–30. 10.1038/nature711 11793011

[60] Zhou J Blundell J Ogawa S Kwon CH Zhang W Sinton C. Pharmacological inhibition of mtorc1 suppresses anatomical, cellular, and behavioral abnormalities in neural-specific pten knock-out mice. J Neurosci. (2009) 29:1773–83. 10.1523/JNEUROSCI.5685-08.2009 19211884 PMC3904448

[61] Goorden SM van Woerden GM van der Weerd L Cheadle JP Elgersma Y. Cognitive deficits in tsc1+/- mice in the absence of cerebral lesions and seizures. Ann Neurol. (2007) 62:648–55. 10.1002/ana.21317 18067135

[62] Ehninger D Han S Shilyansky C Zhou Y Li W Kwiatkowski DJ. Reversal of learning deficits in a tsc2+/- mouse model of tuberous sclerosis. Nat Med. (2008) 14:843–8. 10.1038/nm1788 18568033 PMC2664098

[63] Neves-Pereira M Muller B Massie D Williams JH O'Brien PC Hughes A. Deregulation of eif4e: a novel mechanism for autism. J Med Genet. (2009) 46:759–65. 10.1136/jmg.2009.066852 19556253

[64] Sharma A Hoeffer CA Takayasu Y Miyawaki T McBride SM Klann E. Dysregulation of mtor signaling in fragile x syndrome. J Neurosci. (2010) 30:694–702. 10.1523/JNEUROSCI.3696-09.2010 20071534 PMC3665010

[65] Ehninger D Silva AJ. Genetics and neuropsychiatric disorders: treatment during adulthood. Nat Med. (2009) 15:849–50. 10.1038/nm0809-849 19661989

[66] Ma XM Blenis J. Molecular mechanisms of mtor-mediated translational control. Nat Rev Mol Cell Biol. (2009) 10:307–18. 10.1038/nrm2672 19339977

[67] Wang H Doering LC. Reversing autism by targeting downstream mtor signaling. Front Cell Neurosci. (2013) 7:28. 10.3389/fncel.2013.00028 23533374 PMC3607786

[68] Delgoffe GM Pollizzi KN Waickman AT Heikamp E Meyers DJ Horton MR. The kinase mtor regulates the differentiation of helper t cells through the selective activation of signaling by mtorc1 and mtorc2. Nat Immunol. (2011) 12:295–303. 10.1038/ni.2005 21358638 PMC3077821

[69] Kim MS Kuehn HS Metcalfe DD Gilfillan AM. Activation and function of the mtorc1 pathway in mast cells. J Immunol. (2008) 180:4586–95. 10.4049/jimmunol.180.7.4586 18354181 PMC2698706

[70] Delgoffe GM Kole TP Zheng Y Zarek PE Matthews KL Xiao B. The mtor kinase differentially regulates effector and regulatory t cell lineage commitment. Immunity. (2009) 30:832–44. 10.1016/j.immuni.2009.04.014 19538929 PMC2768135

[71] Deason K Troutman TD Jain A Challa DK Mandraju R Brewer T. Bcap links il-1r to the pi3k-mtor pathway and regulates pathogenic th17 cell differentiation. J Exp Med. (2018) 215:2413–28. 10.1084/jem.20171810 30093533 PMC6122979

[72] Lee K Gudapati P Dragovic S Spencer C Joyce S Killeen N. Mammalian target of rapamycin protein complex 2 regulates differentiation of th1 and th2 cell subsets via distinct signaling pathways. Immunity. (2010) 32:743–53. 10.1016/j.immuni.2010.06.002 20620941 PMC2911434

[73] Xu G Xia J Hua X Zhou H Yu C Liu Z. Activated mammalian target of rapamycin is associated with t regulatory cell insufficiency in nasal polyps. Respir Res. (2009) 10:13. 10.1186/1465-9921-10-13 19250527 PMC2651851

[74] Yu L McPhee CK Zheng L Mardones GA Rong Y Peng J. Termination of autophagy and reformation of lysosomes regulated by mtor. Nature. (2010) 465:942–6. 10.1038/nature09076 20526321 PMC2920749

[75] Pott J Kabat AM Maloy KJ. Intestinal epithelial cell autophagy is required to protect against tnf-induced apoptosis during chronic colitis in mice. Cell Host Microbe. (2018) 23:191–202.e194. 10.1016/j.chom.2017.12.017 29358084

[76] Magdalon J Sánchez-Sánchez SM Griesi-Oliveira K Sertié AL. Dysfunctional mtorc1 signaling: A convergent mechanism between syndromic and nonsyndromic forms of autism spectrum disorder? Int J Mol. Sci. (2017) 18:659. 10.3390/ijms18030659 28335463 PMC5372671

[77] Sato A. Mtor, a potential target to treat autism spectrum disorder. CNS Neurol Disord Drug Targets. (2016) 15:533–43. 10.2174/1871527315666160413120638 27071790 PMC5070418

[78] Burket JA Benson AD Tang AH Deutsch SI. Rapamycin improves sociability in the btbr t(+)itpr3(tf)/j mouse model of autism spectrum disorders. Brain Res Bull. (2014) 100:70–5. 10.1016/j.brainresbull.2013.11.005 24295733 PMC5581959

[79] Chi OZ Wu CC Liu X Rah KH Jacinto E Weiss HR. Restoration of normal cerebral oxygen consumption with rapamycin treatment in a rat model of autism-tuberous sclerosis. Neuromolecular Med. (2015) 17:305–13. 10.1007/s12017-015-8359-5 26048361 PMC4888058

[80] Steinmetz AB Stern SA Kohtz AS Descalzi G Alberini CM. Insulin-like growth factor ii targets the mtor pathway to reverse autism-like phenotypes in mice. J Neurosci. (2018) 38:1015. 10.1523/JNEUROSCI.2010-17.2017 29217683 PMC5783959

[81] Zhang J Zhang JX Zhang QL. Pi3k/akt/mtor-mediated autophagy in the development of autism spectrum disorder. Brain Res Bull. (2016) 125:152–8. 10.1016/j.brainresbull.2016.06.007 27320472

[82] Wu J de Theije CG da Silva SL van der Horst H Reinders MT Broersen LM. Mtor plays an important role in cow's milk allergy-associated behavioral and immunological deficits. Neuropharmacology. (2015) 97:220–32. 10.1016/j.neuropharm.2015.04.035 26027949

[83] Fischer KE Gelfond JAL Soto VY Han C Someya S Richardson A. Health effects of long-term rapamycin treatment: the impact on mouse health of enteric rapamycin treatment from four months of age throughout life. PLoS ONE. (2015) 10:e0126644. 10.1371/journal.pone.0126644 25978367 PMC4433347

[84] Li J Kim SG Blenis J. Rapamycin: one drug, many effects. Cell Metab. (2014) 19:373–9. 10.1016/j.cmet.2014.01.001 24508508 PMC3972801

[85] Kimball SR Jefferson LS. New functions for amino acids: effects on gene transcription and translation. Am J Clin Nutr. (2006) 83:500s. 10.1093/ajcn/83.2.500S 16470021

[86] Shah OJ Anthony JC Kimball SR Jefferson LS. 4e-bp1 and s6k1: translational integration sites for nutritional and hormonal information in muscle. American journal of physiology Endocrinology and metabolism. (2000) 279:E715–729. 10.1152/ajpendo.2000.279.4.E715 11001751

[87] Jewell JL Russell RC Guan K-L. Amino acid signalling upstream of mtor. Nature reviews Molecular cell biology. (2013) 14:133–9. 10.1038/nrm3522 23361334 PMC3988467

[88] Kim J Guan KL. Amino acid signaling in tor activation. Annu. Rev. Biochem. (2011) 80:1001–32. 10.1146/annurev-biochem-062209-094414 21548787

[89] Zheng L Zhang W Zhou Y Li F Wei H Peng J. Recent advances in understanding amino acid sensing mechanisms that regulate mtorc1. Int. J. Mol. Sci. (2016) 17. 10.3390/ijms17101636 27690010 PMC5085669

[90] Dodd KM Tee AR. Leucine and mtorc1: a complex relationship. Am J Physiol Endocrinol Metabol. (2012) 302:E1329–1342. 10.1152/ajpendo.00525.2011 22354780

[91] Goberdhan DC Wilson C Harris AL. Amino acid sensing by mtorc1: intracellular transporters mark the spot. Cell Metab. (2016) 23:580–9. 10.1016/j.cmet.2016.03.013 27076075 PMC5067300

[92] Prizant RL Barash I. Negative effects of the amino acids lys, his, and thr on s6k1 phosphorylation in mammary epithelial cells. J Cell Biochem. (2008) 105:1038–47. 10.1002/jcb.21904 18767117

[93] Wu J de Theije CGM da Silva SL Abbring S van der Horst H Broersen LM. Dietary interventions that reduce mtor activity rescue autistic-like behavioral deficits in mice. Brain Behav Immun. (2017) 59:273–87. 10.1016/j.bbi.2016.09.016 27640900

[94] Ho IC. M(en)tor(ing) differentiating t helper cells. Immunity. (2009) 30:759–61. 10.1016/j.immuni.2009.06.002 19538925

[95] Lee JH Park E Jin HJ. Anti-inflammatory and anti-genotoxic activity of branched chain amino acids (bcaa) in lipopolysaccharide (lps) stimulated raw 264.7 macrophages. Food Sci Biotechnol. (2017) 26:1371–7. 10.1007/s10068-017-0165-4 30263672 PMC6049802

[96] De Theije C Neuroimmunomodulation of the Young Brain. Nutrition, a Gut Feeling. Thesis De Theije, C.G.M. Utrecht University Repository, Utrecht (2014).

[97] Chuang JC Yu CL Wang SR. Modulation of human lymphocyte proliferation by amino acids. Clin Exp Immunol. (1990) 81:173–6. 10.1111/j.1365-2249.1990.tb05310.x 2379319 PMC1535017

[98] Skaper SD Molden DP Seegmiller JE. Maple syrup urine disease: branched-chain amino acid concentrations and metabolism in cultured human lymphoblasts. Biochem Genet. (1976) 14:527–39. 10.1007/BF00485832 985377

[99] Petro TM Bhattacharjee JK. Effect of dietary essential amino acid limitations upon the susceptibility to salmonella typhimurium and the effect upon humoral and cellular immune responses in mice. Infect Immun. (1981) 32:251–9. 7012029 10.1128/iai.32.1.251-259.1981 PMC350614

[100] Novarino G El-Fishawy P Kayserili H Meguid NA Scott EM Schroth J. Mutations in bckd-kinase lead to a potentially treatable form of autism with epilepsy. Science. (2012) 338:394–7. 10.1126/science.1224631 22956686 PMC3704165

[101] Nakamura I. (2014). Impairment of innate immune responses in cirrhotic patients and treatment by branched-chain amino acids. World J Gastroenterol. (2014) 20:7298–305. 10.3748/wjg.v20.i23.7298 24966600 PMC4064075

[102] Vojdani A Mumper E Granpeesheh D Mielke L Traver D Bock K. Low natural killer cell cytotoxic activity in autism: the role of glutathione, il-2 and il-15. J Neuroimmunol. (2008) 205:148–54. 10.1016/j.jneuroim.2008.09.005 18929414

[103] Zhang HW Wang F Wei-zhong L Meng-bin JG Guan C The effects of bcaa-enriched amino acid solution on immune function and protein metabolism in postoperative patients with rectal cancer. Parenter Enteral Nutr. (2007) 2:009 Available online at: http://en.cnki.com.cn/Article_en/CJFDTOTAL-CWCN200702009.htm

[104] Abd El-Aziz SA Alm El-Din RA Cellular-mediated and humoral immunity in children with autism. Egypt J Pediatr Allergy Immunol. (2012) 10:25–32. Available online at: https://www.ajol.info/index.php/ejpai/article/view/108274

[105] Bjorklund G Saad K Chirumbolo S Kern JK Geier DA Geier MR. Immune dysfunction and neuroinflammation in autism spectrum disorder. Acta Neurobiol Exp. (2016) 76:257–68. 10.21307/ane-2017-025 28094817

[106] Bradstreet JJ Sych N Antonucci N Klunnik M Ivankova O Matyashchuk I. Efficacy of fetal stem cell transplantation in autism spectrum disorders: An open-labeled pilot study. Cell Transplant. (2014) 23 (Suppl. 1):S105–12. 10.3727/096368914X684916 25302490

[107] De Simone R Vissicchio F Mingarelli C De Nuccio C Visentin S Ajmone-Cat MA Branched-chain amino acids influence the immune properties of microglial cells and their responsiveness to pro-inflammatory signals. Biochim Biophys Acta. (2013) 1832:650–9. 10.1016/j.bbadis.2013.02.001 23402925

[108] Kim HJ Cho MH Shim WH Kim JK Jeon EY Kim DH. Deficient autophagy in microglia impairs synaptic pruning and causes social behavioral defects. Mol Psychiatry. (2017) 22:1576–84. 10.1038/mp.2016.103 27400854 PMC5658669

[109] Ruth MR Field CJ. The immune modifying effects of amino acids on gut-associated lymphoid tissue. J Anim Sci Biotechnol. (2013) 4:27. 10.1186/2049-1891-4-27 23899038 PMC3750756

[110] Faure M Mettraux C Moennoz D Godin JP Vuichoud J Rochat F. Specific amino acids increase mucin synthesis and microbiota in dextran sulfate sodium-treated rats. J Nutr. (2006) 136:1558–64. 10.1093/jn/136.6.1558 16702321

[111] Liu X Beaumont M Walker F Chaumontet C Andriamihaja M Matsumoto H. Beneficial effects of an amino acid mixture on colonic mucosal healing in rats. Inflamm Bowel Dis. (2013) 19:2895–905. 10.1097/01.MIB.0000435849.17263.c5 24193156

[112] Dawson P Filipe MI Uptake of (3h)thr in human colonic mucosa associated with carcinoma: an autoradiographic analysis at the ultrastructural level. Histocheml J. (1982) 14:385–401. 10.1007/BF01011851 6811506

[113] Remond D Buffiere C Godin JP Mirand PP Obled C Papet I. Intestinal inflammation increases gastrointestinal threonine uptake and mucin synthesis in enterally fed minipigs. J Nutr. (2009) 139:720–6. 10.3945/jn.108.101675 19193812

[114] Hasegawa S Ichiyama T Sonaka I Ohsaki A Hirano R Haneda Y. Amino acids exhibit anti-inflammatory effects in human monocytic leukemia cell line, thp-1 cells. Inflamm Res. (2011) 60:1013–9. 10.1007/s00011-011-0362-1 21785859

[115] Son DO Satsu H Shimizu M. Histidine inhibits oxidative stress- and tnf-alpha-induced interleukin-8 secretion in intestinal epithelial cells. FEBS Lett. (2005) 579:4671–7. 10.1016/j.febslet.2005.07.038 16107255

[116] Andou A Hisamatsu T Okamoto S Chinen H Kamada N Kobayashi T. Dietary histidine ameliorates murine colitis by inhibition of proinflammatory cytokine production from macrophages. Gastroenterology. (2009) 136:564–574.e562. 10.1053/j.gastro.2008.09.062 19027739

[117] Han H Yin J Wang B Huang X Yao J Zheng J. Effects of dietary lysine restriction on inflammatory responses in piglets. Sci Rep. (2018) 8:2451. 10.1038/s41598-018-20689-3 29402921 PMC5799382

[118] Howard A Tahir I Javed S Waring SM Ford D Hirst BH. Glycine transporter glyt1 is essential for glycine-mediated protection of human intestinal epithelial cells against oxidative damage. J Physiol. (2010) 588:995–1009. 10.1113/jphysiol.2009.186262 20123783 PMC2849964

[119] Spittler A Reissner CM Oehler R Gornikiewicz A Gruenberger T Manhart N. Immunomodulatory effects of glycine on lps-treated monocytes: reduced tnf-alpha production and accelerated il-10 expression. FASEB J. (1999) 13:563–71. 10.1096/fasebj.13.3.563 10064624

[120] Ikejima K Iimuro Y Forman DT Thurman RG. A diet containing glycine improves survival in endotoxin shock in the rat. Am J Physiol. (1996) 271:G97–103. 10.1152/ajpgi.1996.271.1.G97 8760112

[121] Ham DJ Murphy KT Chee A Lynch GS Koopman R. Glycine administration attenuates skeletal muscle wasting in a mouse model of cancer cachexia. Clin Nutr. (2014) 33:448–58. 10.1016/j.clnu.2013.06.013 23835111

[122] van Bergenhenegouwen J Braber S Loonstra R Buurman N Rutten L Knipping K Oral exposure to the free amino acid glycine inhibits the onset of cow's milk allergy. Nutr Res. (2018) 58:95–105. 10.1016/j.nutres.2018.07.005 30340819

[123] Ewaschuk JB Murdoch GK Johnson IR Madsen KL Field CJ. Glutamine supplementation improves intestinal barrier function in a weaned piglet model of *escherichia coli* infection. Br J Nutr. (2011) 106:870–7. 10.1017/S0007114511001152 21736826

[124] Vermeulen MA de Jong J Vaessen MJ van Leeuwen PA Houdijk AP. Glutamate reduces experimental intestinal hyperpermeability and facilitates glutamine support of gut integrity. World J Gastroenterol. (2011) 17:1569–73. 10.3748/wjg.v17.i12.1569 21472123 PMC3070128

[125] Wu X Zhang Y Liu Z Li TJ Yin YL. Effects of oral supplementation with glutamate or combination of glutamate and n-carbamylglutamate on intestinal mucosa morphology and epithelium cell proliferation in weanling piglets. J Anim Sci. (2012) 90 (Suppl. 4):337–9. 10.2527/jas.53752 23365372

[126] Zheng HF Wang WQ Li XM Rauw G Baker GB. Body fluid levels of neuroactive amino acids in autism spectrum disorders: a review of the literature. Amino Acids. (2017) 49:57–65. 10.1007/s00726-016-2332-y 27686223 PMC5241332

[127] Guerrant RL Oria RB Moore SR Oria MO Lima AA. Malnutrition as an enteric infectious disease with long-term effects on child development. Nutr Rev. (2008) 66:487–505. 10.1111/j.1753-4887.2008.00082.x 18752473 PMC2562291

[128] Lima NL Soares AM Mota RM Monteiro HS Guerrant RL Lima AA. Wasting and intestinal barrier function in children taking alanyl-glutamine-supplemented enteral formula. J Pediatr Gastroenterol Nutr. (2007) 44:365–74. 10.1097/MPG.0b013e31802eecdd 17325559

[129] Kim MH Kim H. The roles of glutamine in the intestine and its implication in intestinal diseases. Int J Mol Sci. (2017) 18:E1051. 10.3390/ijms18051051 28498331 PMC5454963

[130] Kim H. Glutamine as an immunonutrient. Yonsei Med J. (2011) 52:892–7. 10.3349/ymj.2011.52.6.892 22028151 PMC3220259

[131] Lechowski S Feilhauer K Staib L Coeffier M Bischoff SC Lorentz A. Combined arginine and glutamine decrease release of de novo synthesized leukotrienes and expression of proinflammatory cytokines in activated human intestinal mast cells. Eur J Nutr. (2013) 52:505–12. 10.1007/s00394-012-0353-1 22527286

[132] Pacheco R Ciruela F Casado V Mallol J Gallart T Lluis C. Group i metabotropic glutamate receptors mediate a dual role of glutamate in t cell activation. J Biol Chem. (2004) 279:33352–8. 10.1074/jbc.M401761200 15184389

[133] Pacheco R Oliva H Martinez-Navio JM Climent N Ciruela F Gatell JM. Glutamate released by dendritic cells as a novel modulator of t cell activation. J Immunol. (2006) 177:6695–704. 10.4049/jimmunol.177.10.6695 17082582

[134] Magistretti PJ Pellerin L. Cellular mechanisms of brain energy metabolism and their relevance to functional brain imaging. Philos Trans R Soc Lond Ser B Biol Sci. (1999) 354:1155–63. 10.1098/rstb.1999.0471 10466143 PMC1692634

[135] Sheldon AL Robinson MB. The role of glutamate transporters in neurodegenerative diseases and potential opportunities for intervention. Neurochem Int. (2007) 51:333–55. 10.1016/j.neuint.2007.03.012 17517448 PMC2075474

[136] Cai J Ding L Zhang JS Xue J Wang LZ. Elevated plasma levels of glutamate in children with autism spectrum disorders. Neuroreport. (2016) 27:272–6. 10.1097/WNR.0000000000000532 26825346

[137] El-Ansary A Al-Ayadhi L. Gabaergic/glutamatergic imbalance relative to excessive neuroinflammation in autism spectrum disorders. J Neuroinflammation. (2014) 11:189. 10.1186/s12974-014-0189-0 25407263 PMC4243332

[138] Hassan TH Abdelrahman HM Fattah NRA El-Masry NM Hashim HM El-Gerby KM Blood and brain glutamate levels in children with autistic disorder. Res Autism Spectr Disord. (2013) 7:541–8. 10.1016/j.rasd.2012.12.005

[139] Essa MM Subash S Braidy N Al-Adawi S Lim CK Manivasagam T. Role of nad(+), oxidative stress, and tryptophan metabolism in autism spectrum disorders. Int J Tryptophan Res. (2013) 6:15–28. 10.4137/IJTR.S11355 23922500 PMC3729335

[140] Naushad SM Jain JM Prasad CK Naik U Akella RR. Autistic children exhibit distinct plasma amino acid profile. Indian J Biochem Biophys. (2013) 50:474–8. 24772971

[141] Tirouvanziam R Obukhanych TV Laval J Aronov PA Libove R Banerjee AG. Distinct plasma profile of polar neutral amino acids, leucine, and glutamate in children with autism spectrum disorders. J Autism Dev Disord. (2012) 42:827–36. 10.1007/s10803-011-1314-x 21713591

[142] Sadok I Gamian A Staniszewska MM. Chromatographic analysis of tryptophan metabolites. J Sep Sci. (2017) 40:3020–45. 10.1002/jssc.201700184 28590049 PMC5575536

[143] Koola MM. Kynurenine pathway and cognitive impairments in schizophrenia: Pharmacogenetics of galantamine and memantine. Schizoph Res Cogn. (2016) 4:4–9. 10.1016/j.scog.2016.02.001 27069875 PMC4824953

[144] Bryn V Verkerk R Skjeldal OH Saugstad OD Ormstad H. Kynurenine pathway in autism spectrum disorders in children. Neuropsychobiology. (2017) 76:82–8. 10.1159/000488157 29694960

[145] Lim CK Essa MM de Paula Martins R Lovejoy DB Bilgin AA Waly MI. Altered kynurenine pathway metabolism in autism: implication for immune-induced glutamatergic activity. Autism Res. (2016) 9:621–31. 10.1002/aur.1565 26497015

[146] Fallarino F Grohmann U Vacca C Bianchi R Orabona C Spreca A. T cell apoptosis by tryptophan catabolism. Cell Death Differ. (2002) 9:1069–77. 10.1038/sj.cdd.4401073 12232795

[147] Croonenberghs J Verkerk R Scharpe S Deboutte D Maes M Serotonergic disturbances in autistic disorder: L-5-hydroxytryptophan administration to autistic youngsters increases the blood concentrations of serotonin in patients but not in controls. Life Sci. (2005) 76:2171–83. 10.1016/j.lfs.2004.06.032 15733932

[148] Gabriele S Sacco R Persico AM. Blood serotonin levels in autism spectrum disorder: a systematic review and meta-analysis. Eur Neuropsychopharmacol. (2014) 24:919–29. 10.1016/j.euroneuro.2014.02.004 24613076

[149] Golubeva AV Joyce SA Moloney G Burokas A Sherwin E Arboleya S. Microbiota-related changes in bile acid & tryptophan metabolism are associated with gastrointestinal dysfunction in a mouse model of autism. EBioMedicine. (2017) 24:166–78. 10.1016/j.ebiom.2017.09.020 28965876 PMC5652137

[150] Williams M Zhang Z Nance E Drewes JL Lesniak WG Singh S. Maternal inflammation results in altered tryptophan metabolism in rabbit placenta and fetal brain. Dev Neurosci. (2017) 39:399–412. 10.1159/000471509 28490020 PMC6447288

[151] Kim CJ Kovacs-Nolan JA Yang C Archbold T Fan MZ Mine Y. L-tryptophan exhibits therapeutic function in a porcine model of dextran sodium sulfate (dss)-induced colitis. J Nutr Biochem. (2010) 21:468–75. 10.1016/j.jnutbio.2009.01.019 19428234

[152] Zelante T Iannitti RG Cunha C De Luca A Giovannini G Pieraccini G. Tryptophan catabolites from microbiota engage aryl hydrocarbon receptor and balance mucosal reactivity via interleukin-22. Immunity. (2013) 39:372–85. 10.1016/j.immuni.2013.08.003 23973224

[153] Hashimoto T Perlot T Rehman A Trichereau J Ishiguro H Paolino M. Ace2 links amino acid malnutrition to microbial ecology and intestinal inflammation. Nature. (2012) 487:477–81. 10.1038/nature11228 22837003 PMC7095315

[154] Cryan JF Dinan TG. Mind-altering microorganisms: the impact of the gut microbiota on brain and behaviour. Nat Rev Neurosci. (2012) 13:701–12. 10.1038/nrn3346 22968153

[155] Grenham S Clarke G Cryan JF Dinan TG. Brain-gut-microbe communication in health and disease. Front Physiol. (2011) 2:94. 10.3389/fphys.2011.00094 22162969 PMC3232439

[156] De Palma G Collins SM Bercik P Verdu EF The microbiota-gut-brain axis in gastrointestinal disorders: Stressed bugs, stressed brain or both? J Physiol. (2014) 592:2989–97. 10.1113/jphysiol.2014.273995 24756641 PMC4214655

[157] Forsythe P Sudo N Dinan T Taylor VH Bienenstock J. Mood and gut feelings. Brain Behav Immun. (2010) 24:9–16. 10.1016/j.bbi.2009.05.058 19481599

[158] Huo R Zeng B Zeng L Cheng K Li B Luo Y. Microbiota modulate anxiety-like behavior and endocrine abnormalities in hypothalamic-pituitary-adrenal axis. Front Cell Infect Microbiol. (2017) 7:489. 10.3389/fcimb.2017.00489 29250490 PMC5715198

[159] O'Mahony SM Marchesi JR Scully P Codling C Ceolho AM Quigley EM. Early life stress alters behavior, immunity, and microbiota in rats: implications for irritable bowel syndrome and psychiatric illnesses. Biol Psychiatry. (2009) 65:263–7. 10.1016/j.biopsych.2008.06.026 18723164

[160] Li Q Han Y Dy ABC Hagerman RJ. The gut microbiota and autism spectrum disorders. Front Cell Neurosci. (2017) 11:120–120. 10.3389/fncel.2017.00120 28503135 PMC5408485

[161] Dai ZL Wu G Zhu WY. Amino acid metabolism in intestinal bacteria: links between gut ecology and host health. Front Biosci. (2011) 16:1768–86. 10.2741/3820 21196263

[162] Macfarlane GT Macfarlane S. Bacteria, colonic fermentation, and gastrointestinal health. J AOAC Int. (2012) 95:50–60. 10.5740/jaoacint.SGE_Macfarlane 22468341

[163] Neis EP Dejong CH Rensen SS. The role of microbial amino acid metabolism in host metabolism. Nutrients. (2015) 7:2930–46. 10.3390/nu7042930 25894657 PMC4425181

[164] Macfarlane GT Allison C Gibson SA Cummings JH. Contribution of the microflora to proteolysis in the human large intestine. J Appl Bacteriol. (1988) 64:37–46. 10.1111/j.1365-2672.1988.tb02427.x 3127369

[165] Siniscalco D Schultz S Brigida AL Antonucci N. Inflammation and neuro-immune dysregulations in autism spectrum disorders. Pharmaceuticals. (2018) 11:E56. 10.3390/ph11020056 29867038 PMC6027314

[166] Arnold GL Hyman SL Mooney RA Kirby RS. Plasma amino acids profiles in children with autism: potential risk of nutritional deficiencies. J Autism Dev Disord. (2003) 33:449–54. 10.1023/A:1025071014191 12959424

[167] Evans C Dunstan RH Rothkirch T Roberts TK Reichelt KL Cosford R. Altered amino acid excretion in children with autism. Nutr Neurosci. (2008) 11:9–17. 10.1179/147683008X301360 18510798

[168] Zhang S Zeng X Ren M Mao X Qiao S. Novel metabolic and physiological functions of branched chain amino acids: a review. J Anim Sci Biotechnol. (2017) 8:10. 10.1186/s40104-016-0139-z 28127425 PMC5260006

[169] Booijink C Analysis of Diversity and Function of the Human Small Intestinal Microbiota. Dissertation Booijnk C.C.G.M. Wageningen University, Wageningen (2009).

[170] Finegold SM Dowd SE Gontcharova V Liu C Henley KE Wolcott RD. Pyrosequencing study of fecal microflora of autistic and control children. Anaerobe. (2010) 16:444–53. 10.1016/j.anaerobe.2010.06.008 20603222

[171] Williams BL Hornig M Buie T Bauman ML Cho Paik M Wick I. Impaired carbohydrate digestion and transport and mucosal dysbiosis in the intestines of children with autism and gastrointestinal disturbances. PLoS ONE. (2011) 6:e24585. 10.1371/journal.pone.0024585 21949732 PMC3174969

[172] Williams BL Hornig M Parekh T Lipkin WI. Application of novel pcr-based methods for detection, quantitation, and phylogenetic characterization of sutterella species in intestinal biopsy samples from children with autism and gastrointestinal disturbances. mBio. (2012) 3:e00261–11. 10.1128/mBio.00261-11 22233678 PMC3252763

[173] Bala KA Dogan M Mutluer T Kaba S Aslan O Balahoroglu R. Plasma amino acid profile in autism spectrum disorder (asd). Eur Rev Med Pharmacol Sci. (2016) 20:923–9. 27010152

[174] Davila AM Blachier F Gotteland M Andriamihaja M Benetti PH Sanz Y. Intestinal luminal nitrogen metabolism: role of the gut microbiota and consequences for the host. Pharmacol Res. (2013) 68:95–107. 10.1016/j.phrs.2012.11.005 23183532

[175] Frye RE Rose S Slattery J MacFabe DF. Gastrointestinal dysfunction in autism spectrum disorder: the role of the mitochondria and the enteric microbiome. Microb Ecol Health Dis. (2015) 26:27458. 10.3402/mehd.v26.27458 25956238 PMC4425813

[176] Wang L Christophersen CT Sorich MJ Gerber JP Angley MT Conlon MA. Elevated fecal short chain fatty acid and ammonia concentrations in children with autism spectrum disorder. Dig Dis Sci. (2012) 57:2096–102. 10.1007/s10620-012-2167-7 22535281

[177] MacFabe DF Cain DP Rodriguez-Capote K Franklin AE Hoffman JE Boon F. Neurobiological effects of intraventricular propionic acid in rats: possible role of short chain fatty acids on the pathogenesis and characteristics of autism spectrum disorders. Behav Brain Res. (2007) 176:149–69. 10.1016/j.bbr.2006.07.025 16950524

[178] MacFabe DF Cain NE Boon F Ossenkopp KP Cain DP. Effects of the enteric bacterial metabolic product propionic acid on object-directed behavior, social behavior, cognition, and neuroinflammation in adolescent rats: relevance to autism spectrum disorder. Behav Brain Res. (2011) 217:47–54. 10.1016/j.bbr.2010.10.005 20937326

[179] Ossenkopp KP Foley KA Gibson J Fudge MA Kavaliers M Cain DP. Systemic treatment with the enteric bacterial fermentation product, propionic acid, produces both conditioned taste avoidance and conditioned place avoidance in rats. Behav Brain Res. (2012) 227:134–41. 10.1016/j.bbr.2011.10.045 22085877

[180] Clarke G Stilling RM Kennedy PJ Stanton C Cryan JF Dinan TG. Minireview: gut microbiota: the neglected endocrine organ. Mol Endocrinol. (2014) 28:1221–38. 10.1210/me.2014-1108 24892638 PMC5414803

[181] Gao J Xu K Liu H Liu G Bai M Peng C. Impact of the gut microbiota on intestinal immunity mediated by tryptophan metabolism. Front Cell Infect Microbiol. (2018) 8:13. 10.3389/fcimb.2018.00013 29468141 PMC5808205

[182] Anderson G Maes M Berk M. Inflammation-related disorders in the tryptophan catabolite pathway in depression and somatization. Adv Protein Chem Struct Biol. (2012) 88:27–48. 10.1016/B978-0-12-398314-5.00002-7 22814705

[183] Nikolaus S Schulte B Al-Massad N Thieme F Schulte DM Bethge J. Increased tryptophan metabolism is associated with activity of inflammatory bowel diseases. Gastroenterology. (2017) 153:1504–1516.e1502. 10.1053/j.gastro.2017.08.028 28827067

[184] Inoue R Sakaue Y Sawai C Sawai T Ozeki M Romero-Perez GA. A preliminary investigation on the relationship between gut microbiota and gene expressions in peripheral mononuclear cells of infants with autism spectrum disorders. Biosci Biotechnol Biochem. (2016) 80:2450–8. 10.1080/09168451.2016.1222267 27581276

[185] Strati F Cavalieri D Albanese D De Felice C Donati C Hayek J. New evidences on the altered gut microbiota in autism spectrum disorders. Microbiome. (2017) 5:24. 10.1186/s40168-017-0242-1 28222761 PMC5320696

[186] Tomova A Husarova V Lakatosova S Bakos J Vlkova B Babinska K. Gastrointestinal microbiota in children with autism in slovakia. Physiol Behav. (2015) 138:179–87. 10.1016/j.physbeh.2014.10.033 25446201

[187] Ley RE Turnbaugh PJ Klein S Gordon JI. Microbial ecology: human gut microbes associated with obesity. Nature. (2006) 444:1022–3. 10.1038/4441022a 17183309

[188] Shen X Miao J Wan Q Wang S Li M Pu F. Possible correlation between gut microbiota and immunity among healthy middle-aged and elderly people in southwest china. Gut Pathog. (2018) 10:4. 10.1186/s13099-018-0231-3 29449892 PMC5806246

[189] Zhou Y Zhi F. Lower level of bacteroides in the gut microbiota is associated with inflammatory bowel disease: a meta-analysis. Biomed Res Int. (2016) 2016:5828959. 10.1155/2016/5828959 27999802 PMC5143693

[190] Ren W Chen S Yin J Duan J Li T Liu G. Dietary arginine supplementation of mice alters the microbial population and activates intestinal innate immunity. J Nutr. (2014) 144:988–95. 10.3945/jn.114.192120 24670969

[191] Zhang Y Lu T Han L Zhao L Niu Y Chen H. L-glutamine supplementation alleviates constipation during late gestation of mini sows by modifying the microbiota composition in feces. Biomed Res Int. (2017) 2017:4862861. 10.1155/2017/4862861 28386552 PMC5366184

[192] Rizzatti G Lopetuso LR Gibiino G Binda C Gasbarrini A. Proteobacteria: a common factor in human diseases. Biomed Res. Int. (2017) 2017:9351507–9351507. 10.1155/2017/9351507 29230419 PMC5688358

[193] Shin NR Whon TW Bae JW. Proteobacteria: microbial signature of dysbiosis in gut microbiota. Trends Biotechnol. (2015) 33:496–503. 10.1016/j.tibtech.2015.06.011 26210164

[194] Yang Z Huang S Zou D Dong D He X Liu N. Metabolic shifts and structural changes in the gut microbiota upon branched-chain amino acid supplementation in middle-aged mice. Amino Acids. (2016) 48:2731–45. 10.1007/s00726-016-2308-y 27539648

[195] Vojdani A Lambert J Vojdani E Blood-based markers in autism spectrum disorders. Int Med Rev. (2017) 3:1–30. Available online at: https://www.researchgate.net/publication/321642638_Blood-based_markers_in_autism_spectrum_disorders_Blood-based_markers_in_autism_spectrum_disorders

